# Plasma Microbial Cell-Free DNA Sequencing in Immunocompromised Patients With Pneumonia: A Prospective Observational Study

**DOI:** 10.1093/cid/ciad599

**Published:** 2023-10-10

**Authors:** Stephen P Bergin, Roy F Chemaly, Sanjeet S Dadwal, Joshua A Hill, Yeon Joo Lee, Ghady Haidar, Alfred Luk, Alexander Drelick, Peter V Chin-Hong, Esther Benamu, Fareed Khawaja, Deepa Nanayakkara, Genovefa A Papanicolaou, Catherine Butkus Small, Monica Fung, Michelle A Barron, Thomas Davis, Micah T McClain, Eileen K Maziarz, Deng B Madut, Armando D Bedoya, Daniel L Gilstrap, Jamie L Todd, Christina E Barkauskas, Robert Bigelow, Jeffrey D Leimberger, Ephraim L Tsalik, Olivia Wolf, Mona Mughar, Desiree Hollemon, Radha Duttagupta, Daniel S Lupu, Sivan Bercovici, Bradley A Perkins, Timothy A Blauwkamp, Vance G Fowler, Thomas L Holland

**Affiliations:** Department of Medicine, Division of Pulmonary, Allergy, and Critical Care Medicine, Duke University School of Medicine, Durham, North Carolina, USA; Duke Clinical Research Institute, Durham, North Carolina, USA; Department of Infectious Diseases, Infection Control and Employee Health, The University of Texas MD Anderson Cancer Center, Houston, Texas, USA; Department of Medicine, Division of Infectious Diseases, City of Hope National Medical Center, Duarte California, California, USA; Vaccine and Infectious Disease Division, Fred Hutchinson Cancer Center, Seattle, Washington, USA; Department of Medicine, University of Washington School of Medicine, Seattle, Washington, USA; Infectious Diseases Service, Memorial Sloan Kettering Cancer Center, NewYork, New York, USA; Department of Medicine, Weill Cornell Medicine, NewYork, New York, USA; Department of Medicine, Division of Infectious Diseases, University of Pittsburgh Medical Center, Pittsburgh, Pennsylvania, USA; Section of Infectious Diseases, John W. Deming Department of Medicine, Tulane University School of Medicine, New Orleans, Louisiana, USA; Department of Medicine, Weill Cornell Medicine, NewYork, New York, USA; Department of Medicine, NewYork-Presbyterian Hospital, New York, New York, USA; Division of Infectious Diseases, University of California San Francisco, San Francisco, California, USA; Division of Infectious Diseases, University of Colorado, Aurora, Colorado, USA; Department of Infectious Diseases, Infection Control and Employee Health, The University of Texas MD Anderson Cancer Center, Houston, Texas, USA; Department of Medicine, Division of Infectious Diseases, City of Hope National Medical Center, Duarte California, California, USA; Infectious Diseases Service, Memorial Sloan Kettering Cancer Center, NewYork, New York, USA; Department of Medicine, Weill Cornell Medicine, NewYork, New York, USA; Department of Medicine, Weill Cornell Medicine, NewYork, New York, USA; Department of Medicine, NewYork-Presbyterian Hospital, New York, New York, USA; Division of Infectious Diseases, University of California San Francisco, San Francisco, California, USA; Division of Infectious Diseases, University of Colorado, Aurora, Colorado, USA; Department of Pathology and Laboratory Medicine, Indiana University School of Medicine, Indianapolis, Indiana, USA; Duke Clinical Research Institute, Durham, North Carolina, USA; Department of Medicine, Division of Infectious Diseases, Duke University School of Medicine, Durham, North Carolina, USA; Department of Medicine, Division of Infectious Diseases, Duke University School of Medicine, Durham, North Carolina, USA; Department of Medicine, Division of Infectious Diseases, Duke University School of Medicine, Durham, North Carolina, USA; Department of Medicine, Division of Pulmonary, Allergy, and Critical Care Medicine, Duke University School of Medicine, Durham, North Carolina, USA; Department of Medicine, Division of Pulmonary, Allergy, and Critical Care Medicine, Duke University School of Medicine, Durham, North Carolina, USA; Department of Medicine, Division of Pulmonary, Allergy, and Critical Care Medicine, Duke University School of Medicine, Durham, North Carolina, USA; Duke Clinical Research Institute, Durham, North Carolina, USA; Department of Medicine, Division of Pulmonary, Allergy, and Critical Care Medicine, Duke University School of Medicine, Durham, North Carolina, USA; Duke Clinical Research Institute, Durham, North Carolina, USA; Duke Clinical Research Institute, Durham, North Carolina, USA; Department of Medicine, Division of Infectious Diseases, Duke University School of Medicine, Durham, North Carolina, USA; Emergency Medicine Services, Durham Veterans Affairs Health Care System, Durham, North Carolina, USA; VP and Chief Scientific Officer, Infectious Disease, Danaher Diagnostics, Washington, DC, USA; Duke Clinical Research Institute, Durham, North Carolina, USA; Karius Inc., Redwood City, California, USA; Karius Inc., Redwood City, California, USA; Karius Inc., Redwood City, California, USA; Karius Inc., Redwood City, California, USA; Karius Inc., Redwood City, California, USA; Karius Inc., Redwood City, California, USA; Karius Inc., Redwood City, California, USA; Duke Clinical Research Institute, Durham, North Carolina, USA; Department of Medicine, Division of Infectious Diseases, Duke University School of Medicine, Durham, North Carolina, USA; Duke Clinical Research Institute, Durham, North Carolina, USA; Department of Medicine, Division of Infectious Diseases, Duke University School of Medicine, Durham, North Carolina, USA

**Keywords:** immunocompromised pneumonia, bronchoscopy, hematologic malignancy, hematopoietic cell transplant, microbial cell-free DNA sequencing

## Abstract

**Background:**

Pneumonia is a common cause of morbidity and mortality, yet a causative pathogen is identified in a minority of cases. Plasma microbial cell-free DNA sequencing may improve diagnostic yield in immunocompromised patients with pneumonia.

**Methods:**

In this prospective, multicenter, observational study of immunocompromised adults undergoing bronchoscopy to establish a pneumonia etiology, plasma microbial cell-free DNA sequencing was compared to standardized usual care testing. Pneumonia etiology was adjudicated by a blinded independent committee. The primary outcome, additive diagnostic value, was assessed in the Per Protocol population (patients with complete testing results and no major protocol deviations) and defined as the percent of patients with an etiology of pneumonia exclusively identified by plasma microbial cell-free DNA sequencing. Clinical additive diagnostic value was assessed in the Per Protocol subgroup with negative usual care testing.

**Results:**

Of 257 patients, 173 met Per Protocol criteria. A pneumonia etiology was identified by usual care in 52/173 (30.1%), plasma microbial cell-free DNA sequencing in 49/173 (28.3%) and the combination of both in 73/173 (42.2%) patients. Plasma microbial cell-free DNA sequencing exclusively identified an etiology of pneumonia in 21/173 patients (additive diagnostic value 12.1%, 95% confidence interval [CI], 7.7% to 18.0%, *P* < .001). In the Per Protocol subgroup with negative usual care testing, plasma microbial cell-free DNA sequencing identified a pneumonia etiology in 21/121 patients (clinical additive diagnostic value 17.4%, 95% CI, 11.1% to 25.3%).

**Conclusions:**

Non-invasive plasma microbial cell-free DNA sequencing significantly increased diagnostic yield in immunocompromised patients with pneumonia undergoing bronchoscopy and extensive microbiologic and molecular testing.

**Clinical Trials Registration:**

NCT04047719.

## Teaser

In this prospective multicenter observational study of immunocompromised patients undergoing bronchoscopy, plasma microbial cell-free DNA sequencing exclusively identified an adjudicated pneumonia etiology in 12.1% of patients with otherwise nondiagnostic testing, significantly increasing overall diagnostic yield in this high-risk population.

## Graphical Abstract

**Graphical Abstract:**
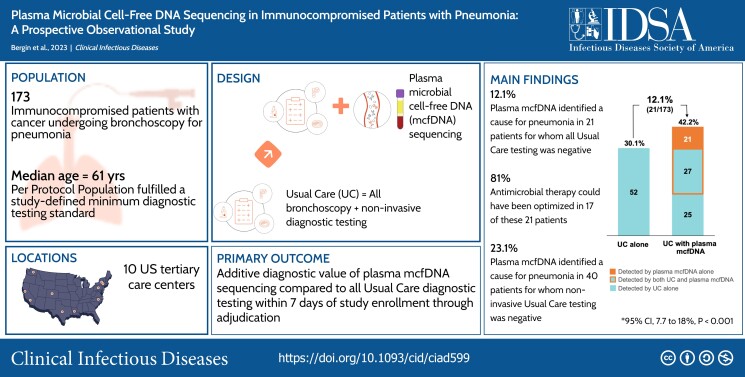
This graphical abstract is also available at Tidbit: https://tidbitapp.io/tidbits/plasma-microbial-cell-free-dna-sequencing-in-immunocompromised-patients-with-pneumonia-aprospective-observational-study-bb13c9de-87ac-430c-a84e-6fe9f3f79932

**(See the Editorial Commentary by Hanson and Caliendo on pages 785–7.)**

Pneumonia is a common and often lethal complication of hematologic malignancy and hematopoietic cell transplantation [1, 2]. Promptly identifying a causative pathogen can reduce time to effective antimicrobial therapy, yet current diagnostic standards fail to identify an etiology in most cases [2–4]. Bronchoscopy is commonly performed in these patients, but utility is limited when this costly procedure is delayed, and complications occur in approximately 10% [5–7].

New strategies are needed to increase diagnostic yield and reduce complications from either invasive diagnostic procedures or unnecessary treatments. Plasma microbial cell-free DNA (mcfDNA) metagenomic sequencing may offer a non-invasive means of improving diagnostic yield and reducing time to pathogen detection, as compared to current testing strategies reliant upon a battery of microbiologic and molecular tests. In previous retrospective studies of immunocompromised patients with pneumonia, plasma mcfDNA sequencing detected etiologies otherwise identifiable only by invasive diagnostics and, in some cases, causative pathogens where usual care testing was negative altogether [8–11].

The Pneumonia in the Immunocompromised—Use of the Karius Test for the Detection of Undiagnosed Pathogens (PICKUP) study was designed to prospectively evaluate the additive diagnostic value and clinical impact of adding plasma mcfDNA sequencing to usual care testing in significantly immunocompromised patients undergoing bronchoscopy to establish a microbiologic etiology of pneumonia.

## METHODS

### Study Design and Participants

This multicenter, prospective, observational study was conducted at 10 tertiary care centers in the United States. Hospitalized adult patients undergoing diagnostic bronchoscopy to establish a microbiologic etiology of clinically suspected pneumonia were eligible for enrollment if they were receiving treatment for an active hematologic malignancy, recently underwent hematopoietic cell transplantation, or were receiving immunosuppressive therapy for active graft versus host disease. Eligible patients underwent bronchoscopy no more than 1 day prior to enrollment or were scheduled for bronchoscopy within 5 days of enrollment. Patients with an established microbiologic etiology of pneumonia prior to screening or any positive molecular test for severe acute respiratory syndrome coronavirus 2 in the previous 14 days were excluded. The complete study protocol is provided as Supplementary materials.

Clinician documentation and results of all imaging, microbiologic, molecular, and serologic testing performed per usual care from 3 days before through 14 days after enrollment were recorded. Study blood and nasopharyngeal swab samples collected within 24 hours of enrollment, and remnant bronchoalveolar lavage fluid were preserved and sent for supplemental testing at a centralized reference laboratory (Indiana University Core laboratory) when protocol-specified diagnostic testing was not performed as part of usual care. Patients were followed for 50 days to capture final culture or reference laboratory testing results, key clinical outcomes, and antimicrobial exposure through study completion.

### Plasma Microbial Cell-Free DNA Sequencing Analysis

The Karius Test® was developed and validated to detect and quantify mcfDNA in plasma. A detailed description of test methods and validation has been previously described [9]. An overview of plasma mcfDNA sequencing test procedures and an example clinical report are provided in Supplementary materials. Results of plasma mcfDNA sequencing were not provided to members of the clinical team caring for enrolled patients.

### Study Definitions

Usual care testing was defined as the protocol-required minimum diagnostic standard plus any additional diagnostic testing performed during routine care to establish a pathogenic cause of pneumonia. The minimum diagnostic standard predefined a minimum set of bronchoscopic and non-invasive diagnostic tests required for inclusion in the Per Protocol population (Supplementary Figure 1 in Supplementary materials). Protocol-specified testing from bronchoalveolar lavage fluid included bacterial, fungal, and mycobacterial cultures, as well as staining or molecular testing for *Pneumocystis jirovecii.* Non-invasive tests required to complete the minimum diagnostic standard included blood culture, serum galactomannan, and a multiplex respiratory viral panel from nasopharyngeal swab or wash. The Per Protocol population included patients with complete minimum diagnostic standard testing, a valid plasma mcfDNA sequencing test (defined as a specimen processed per protocol and passing internal quality control assessments) collected within 24 hours of enrollment, and no protocol deviations that might bias a comparison of usual care to plasma mcfDNA sequencing test results. Probable cause of pneumonia was defined as a microbe identified by usual care or plasma mcfDNA sequencing adjudicated as a cause of the index pneumonia event following review of all diagnostic testing and clinical information.

### Primary and Secondary Objectives

The primary study objective was to evaluate the additive diagnostic value of plasma mcfDNA sequencing in the Per Protocol population, as compared to an adjudicated composite of all usual care testing results available within 7 days of study enrollment. Key secondary objectives included estimates of the additive diagnostic value of plasma mcfDNA sequencing versus composites of all invasive and noninvasive usual care testing, respectively, and agreement between plasma mcfDNA sequencing and an adjudicated composite of all usual care diagnostic testing. To conservatively estimate agreement with a composite of all usual care testing, RNA viruses (which are not detected by plasma mcfDNA sequencing) were included in agreement analyses.

The primary outcome, additive diagnostic value, was defined as the percent of patients with a probable cause of pneumonia exclusively identified by plasma mcfDNA sequencing. Additive diagnostic value estimates the expected increase in diagnostic yield if plasma mcfDNA sequencing were to be routinely combined with usual care testing in this immunocompromised population. Clinical additive diagnostic value was defined as the percent of patients with an etiology of pneumonia exclusively identified by plasma mcfDNA sequencing in the Per Protocol population subgroup with negative usual care testing. Clinical additive diagnostic value estimates the probability that plasma mcfDNA sequencing would identify a cause of pneumonia in cases where usual care testing was negative.

### Endpoint Adjudication Process

A centralized committee of 4 Infectious Diseases (D. M., E. M., M. M., T. H.) and 4 Pulmonary (A. B., C. B., D. G., J. T.) physicians with significant clinical experience caring for immunocompromised patients with pneumonia adjudicated all study endpoints in a 2-step process. Adjudicators blinded to plasma mcfDNA sequencing results first reviewed a composite of all clinical documentation and usual care diagnostic testing available to determine if a probable cause of the index pneumonia event was identified within 7 days of study enrollment. Results of plasma mcfDNA sequencing were then unblinded and adjudicated. All microbes listed on the plasma mcfDNA sequencing report were classified as either (1) a probable cause of the index pneumonia event, (2) a probable cause of a clinically relevant infection other than pneumonia, or (3) not causing an active infection (commensal organism or contaminant). Adjudicators then determined whether plasma mcfDNA sequencing results were concordant with usual care testing. When a probable cause of pneumonia was identified by plasma mcfDNA sequencing, adjudicators determined whether the diagnostic information would likely change clinical management had the results been available in real time. Each case was independently reviewed by an Infectious Diseases specialist and Pulmonologist. Disagreements between primary adjudications were resolved by review of a committee comprising at least three physicians who had not performed the primary adjudications. The committee was also blinded to plasma mcfDNA sequencing results until usual care adjudication was complete. Additional details of the Clinical Events Classification adjudication process are provided in Supplementary materials.

### Statistical Methods

The study minimum sample size (169) was determined by additive diagnostic value, relative to the Per Protocol population, that would provide 80% power to reject the null hypothesis that ≤5% of patients would have a probable cause of pneumonia exclusively identified by plasma mcfDNA sequencing assuming an additive diagnostic value of ≥10%, with 1-sided type I error =0.05. A non-binding interim test for futility was conducted after the first 56 Per Protocol patients were evaluated. Additive diagnostic value is reported as percent with two-sided Clopper-Pearson exact 95% binomial confidence limits. SAS version 9.4 (SAS Institute Inc.) was used for all statistical analyses.

## RESULTS

### Patients

**Figure 1.**
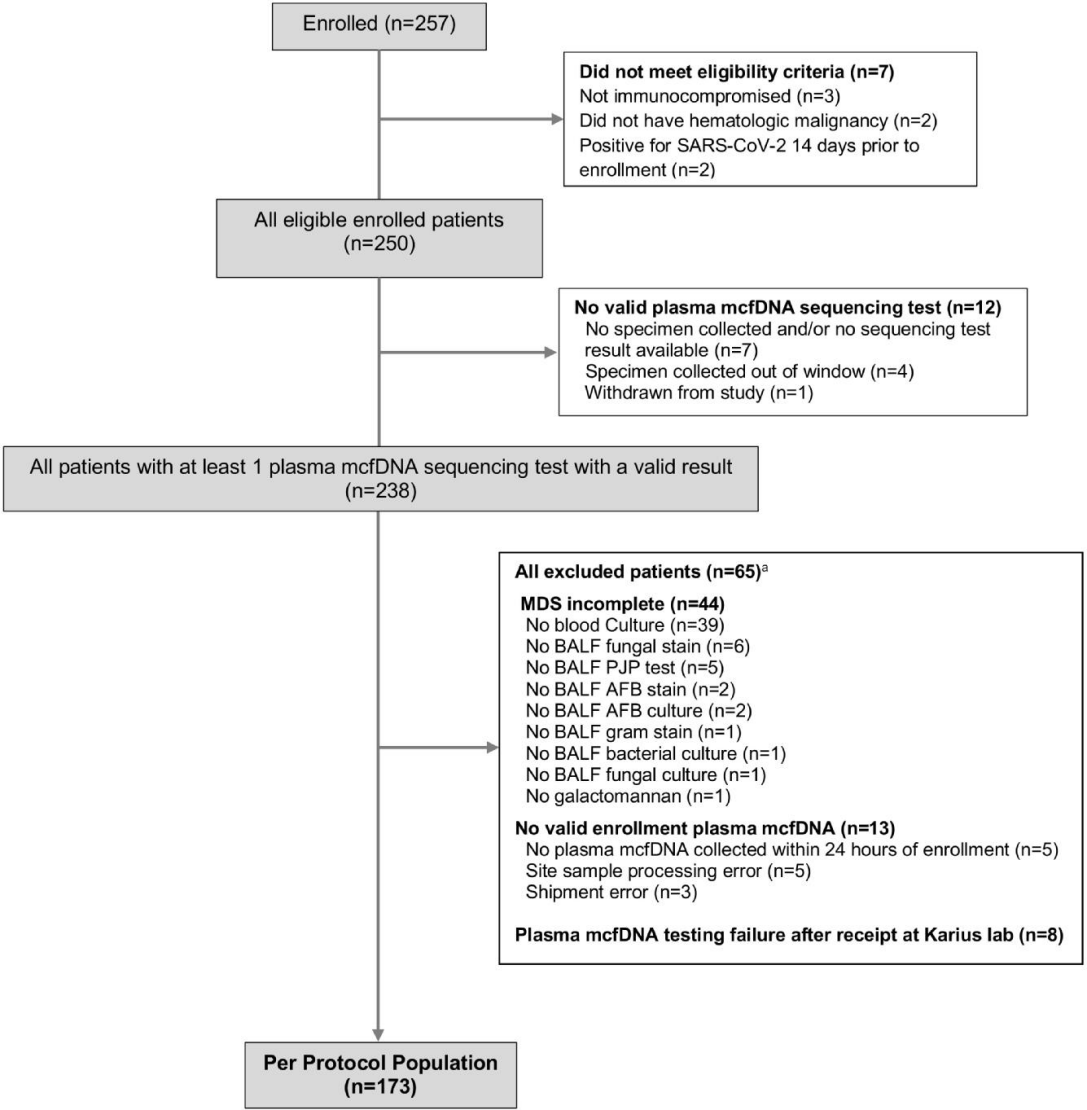
Enrollment and study cohorts. Abbreviations: AFB, acid-fast bacillus; BALF, bronchoalveolar lavage fluid; DNA, deoxyribonucleic acid; mcfDNA, microbial cell-free DNA; MDS, minimum diagnostic standard; PJP, *Pneumocystis jirovecii* pneumonia; SARS-CoV-2, severe acute respiratory syndrome coronavirus 2. ^a^Overlapping categories. Some patients had >1 reason for exclusion.

From 3 January 2020, to 4 February 2022, 257 patients were enrolled from 10 centers with 250 (97.3%) meeting all study eligibility criteria (Figure 1). Of these, 190/250 (76.0%) patients had received chemotherapy within 45 days of enrollment; 138/250 (55.2%) had active relapsed hematologic malignancy, and 75/250 (30.0%) had undergone hematopoietic cell transplantation (Table 1). In the 7 days prior to enrollment, 232 (93.2%) patients received broad antibacterial and 177 (71.1%) received mold-active antifungal therapy. Overall mortality at the final study assessment (50 days after enrollment) was 62/250 (24.8%). Among 247 patients undergoing ≥1 diagnostic bronchoscopy, at least 1 bronchoscopy complication was observed in 40 (16.2%). Life-threatening complications (respiratory failure requiring intensive care unit [ICU] admission and unstable cardiac dysrhythmias) occurred in 5/247 (2.0%) patients. Of 250 patients, 173 (69.2%) met criteria for inclusion in the Per Protocol population. Incomplete minimum diagnostic standard testing, primarily missing blood cultures in 39/250 (15.6%) patients, was the most common reason for exclusion. Baseline characteristics of patients in all study populations were similar to the Per Protocol population.

**Table 1.** Characteristics and Clinical Outcomes of Enrolled Patients

Characteristic	All Eligible Enrolled Patients (n = 250)	Intent-to-Diagnose Population^a^ (n = 238)	Per Protocol Population^b^ (n = 173)
Age—years			
Median (IQR)	62.0 (50.0, 69.0)	62.0 (50.0, 69.0)	61.0 (47.0, 68.0)
Range	21–90	21–90	21–90
Male sex, no. (%)^c^	166/249 (66.7)	159/238 (66.8)	113/173 (65.3)
Ethnicity, no. (%)^c,d^			
Hispanic or Latino	32/236 (13.6)	32/226 (14.2)	21/165 (12.7)
Race, no. (%)^c,d^			
White	199/235 (84.7)	191/225 (84.9)	134/163 (82.2)
Asian	19/235 (8.1)	17/225 (7.6)	16/163 (9.8)
Black or African American	15/235 (6.4)	15/225 (6.7)	11/163 (6.7)
Native Hawaiian or Pacific Islander	2/235 (0.9)	2/225 (0.9)	2/163 (1.2)
Hematologic malignancy type^e^			
Leukemia, no. (%)	166 (66.4)	159 (66.8)	118 (68.2)
Lymphoma, no. (%)	49 (19.6)	45 (18.9)	33 (19.1)
Myelodysplastic syndrome, no. (%)	42 (16.8)	40 (16.8)	29 (16.8)
Multiple myeloma, no. (%)	22 (8.8)	22 (9.2)	15 (8.7)
Prior hematopoietic cell transplant, no. (%)	75 (30.0)	70 (29.4)	55 (31.8)
Allogeneic	66/75 (88.0)	61/70 (87.1)	49/55 (89.1)
Autologous	9/75 (12.0)	9/70 (12.9)	6/55 (10.9)
Relapsed hematologic malignancy at enrollment, no. (%)	140 (56.0)	139 (58.4)	109 (63.0)
Chemotherapy received within 45 d of enrollment, no. (%)	193 (77.2)	185 (77.7)	136 (78.6)
Prior anti-infective therapy at enrollment and up to 7 d prior, no. (%)^c^	249/249 (100)	238 (100)	173 (100)
Anti-Pseudomonal antibacterial	232/249 (93.2)	222/238 (93.3)	164/173 (94.8)
Anti-MRSA antibacterial	162/249 (65.1)	157/238 (66.0)	118/173 (68.2)
Mold-active antifungal	177/249 (71.1)	169/238 (71.0)	129/173 (74.6)
*Pneumocystis jirovecii* prophylaxis	59/249 (23.7)	57/238 (23.9)	41/173 (23.7)
Invasive diagnostic procedures, 1 d prior through 5 d after enrollment, no. (%)^f^	247 (98.8)	236 (99.2)	173 (100)
Bronchoscopy	247/247 (100)	236/236 (100)	173/173 (100)
Other invasive procedures	11/247 (4.5)	11/236 (4.7)	8/173 (4.6%)
Thoracentesis	6/247 (2.4)	6/236 (2.5)	4/173 (2.3)
Transthoracic lung needle aspiration	3/247 (1.2)	3/236 (1.3)	1/173 (0.6)
Surgical lung biopsy	0/247 (0)	0/236 (0)	0/173 (0)
Complications from bronchoscopy performed 1 d prior through 5 d after enrollment, no. (%)^g^			
Any complication	40/247 (16.2)	39/236 (16.5)	26/173 (15.0)
New/worsened hypoxemia	22/247 (8.9)	21/236 (8.9)	13/173 (7.5)
Bronchospasm	9/247 (3.6)	9/236 (3.8)	5/173 (2.9)
Cough	6/247 (2.4)	6/236 (2.5)	5/173 (2.9)
Airway bleeding	5/247 (2.0)	5/236 (2.1)	4/173 (2.3)
Respiratory failure requiring ICU admission/transfer	3/247 (1.2)	3/236 (1.3)	3/173 (1.7)
Shortness of breath	3/247 (1.2)	3/236 (1.3)	2/173 (1.2)
Cardiac arrhythmia requiring treatment	2/247 (0.8)	2/236 (0.8)	2/173 (1.2)
Chest pain	2/247 (0.8)	2/236 (0.8)	2/173 (1.2)
Overall study mortality, no. (%)^h^	62 (24.8)	59 (24.8)	47 (27.2)

Abbreviations: DNA, deoxyribonucleic acid; ICU, intensive care unit; IQR, interquartile range; mcfDNA, microbial cell-free DNA; MRSA, methicillin-resistant *Staphylococcus aureus*.

^a^The Intent to Diagnose population included all eligible patients with a valid plasma microbial cell-free DNA sequencing test.

^b^The Per Protocol population included patients with complete protocol-required diagnostic testing, a valid plasma mcfDNA sequencing test collected within 24 hours of enrollment, and no major protocol deviations.

^c^Characteristic not reported for some patients. The denominator indicates the number of patients in which the characteristic was recorded.

^d^Race and ethic group were self-reported.

^e^Some patients had ≥1 type of active or prior hematologic malignancy.

^f^Includes invasive diagnostic procedures performed to establish pneumonia etiology. Some patients underwent ≥1 diagnostic procedure.

^g^Includes bronchoscopy complications occurring within 24 hours of the procedure.

^h^Includes patients who died the day of or before the end of study visit (median 51 days after enrollment).

### Pneumonia Diagnostic Outcome

At least 1 adjudicated probable cause of pneumonia was identified by usual care testing within 7 days of study enrollment in 52/173 (30.1%) patients in the Per Protocol population (Supplementary Table 1 in Supplement Materials). Of these, 2 probable causes of pneumonia were identified in 3/52 (5.8%) patients. Among 55 probable causes of pneumonia, 30 (54.5%) fungal, 18 (32.7%) bacterial, 3 (5.5%) DNA viral, and 4 (7.3%) RNA viral pathogens were identified by usual care testing. *Aspergillus* species were adjudicated as a causative pathogen in 21/30 (70.0%) patients with fungal pneumonia, of which the diagnosis was based on galactomannan and imaging criteria in 16/21 (76.2%). Post hoc polymerase chain reaction (PCR) testing for *Aspergillus* species was performed on remnant bronchoalveolar lavage fluid in these patients (Suppplementary Table 2 in Supplement Materials). A probable cause of pneumonia was identified by non-invasive usual care testing in 13/173 (7.5%) patients. Plasma mcfDNA sequencing identified at least 1 probable cause of pneumonia in 49/173 (28.3%) patients. Among 56 adjudicated probable causes of pneumonia, plasma mcfDNA sequencing identified 23 (41.1%) fungi, 29 (51.8%) bacteria, and 4 (7.1%) DNA viruses.

### Additive Diagnostic Value

**Figure 2.**
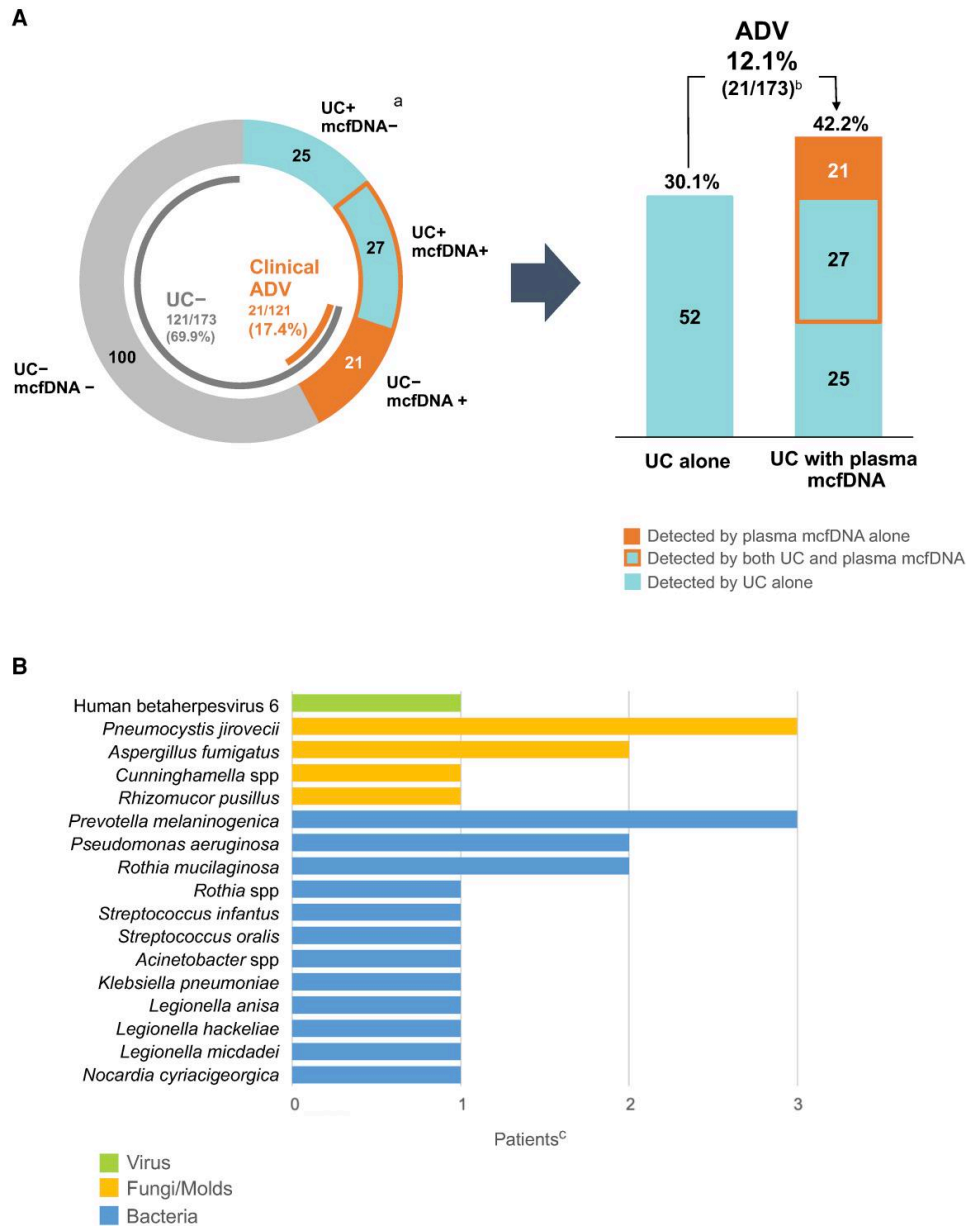
Additive diagnostic value of plasma microbial cell-free DNA dequencing. *A*, Pneumonia diagnostic outcome. *B*, Pneumonia etiologies exclusively identified by plasma mcfDNA sequencing. Abbreviations: ADV, additive diagnostic value; CI, confidence interval; DNA, deoxyribonucleic acid; mcfDNA, microbial cell-free DNA; UC, usual care. ^a^Includes 4 RNA viruses that cannot be detected by plasma microbial cell-free DNA sequencing. ^b^Additive diagnostic value, defined as the percentage of patients with an etiology of pneumonia exclusively identified by plasma microbial cell-free DNA sequencing in the Per Protocol population, was 21/173 = 12.1% (95%CI, 7.7% to 18.0%). An etiology of pneumonia was identified by usual care in 52/173 (30.1%) patients and the combination of usual care and plasma microbial cell-free DNA sequencing in 73/173 (42.2%) patients, equating to a 40.2% relative increase in diagnostic yield when plasma microbial cell-free DNA sequencing was added to usual care testing. ^c^Three patients had 2 adjudicated probable causes of pneumonia detected by plasma mcfDNA sequencing.

**Figure 3.**
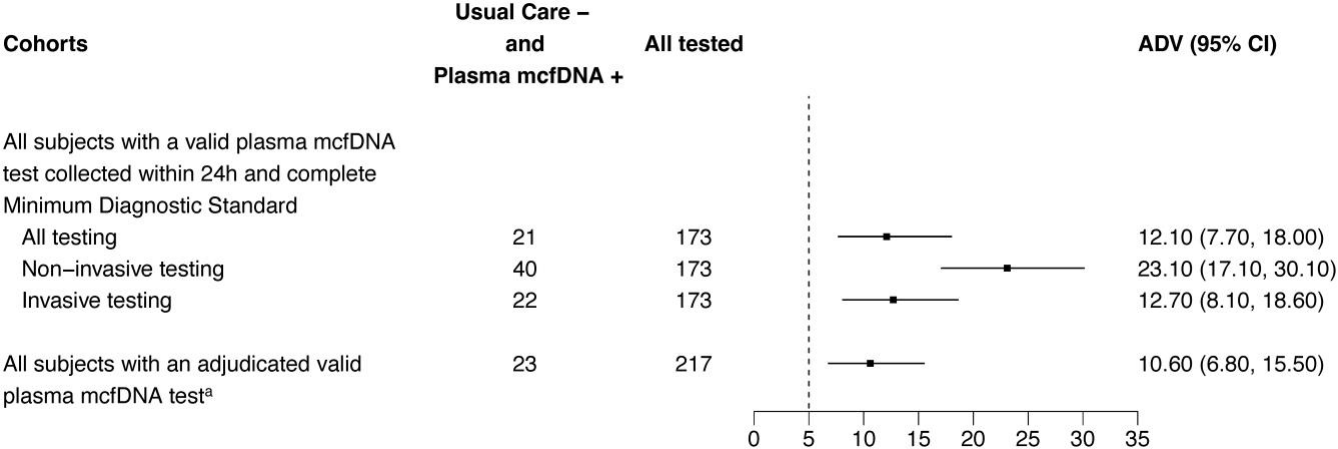
Additive diagnostic value of plasma microbial cell-free DNA sequencing in key study subgroups. Abbreviations: ADV, additive diagnostic value; CI, confidence interval; DNA, deoxyribonucleic acid; mcfDNA, microbial cell-free DNA. ^a^Excludes 21 patients with plasma microbial cell-free DNA sequencing test results that were not adjudicated because the sample was either collected outside the protocol-specified testing window or incorrectly processed at the enrolling site.

In the Per Protocol population, plasma mcfDNA sequencing exclusively identified a probable cause of pneumonia in 21/173 patients for an additive diagnostic value of 12.1% (95% confidence interval [CI], 7.7% to 18.0%, *P* < .001) (Figure 2*A*). The combination of plasma mcfDNA sequencing and usual care testing identified a probable cause of pneumonia in 73/173 (42.2%) patients, equating to a 12.1% absolute increase in diagnostic yield and number needed to test to identify an additional cause of pneumonia of 8.3 (95% CI, 5.6 to 13.0) patients. Among 121 patients with no cause of pneumonia identified by usual care testing, the 21 patients with a probable cause of pneumonia exclusively identified by plasma mcfDNA sequencing represented a clinical additive diagnostic value of 17.4% (95% CI, 11.1% to 25.3%). Additive diagnostic value was similar in prespecified key subgroups and in all enrolled patients with a valid plasma microbial cell-free DNA sequencing result (Figure 3).

### Potential Clinical Impact of Plasma MCFDNA Sequencing

Within the 21 patients with a probable cause of pneumonia exclusively identified by plasma mcfDNA sequencing, 24 causative pathogens were identified including 16 (67%) bacteria, 7 (29%) fungi, and 1 (4%) DNA virus (Figure 2*B*). The adjudication committee determined that plasma mcfDNA sequencing results could have changed antimicrobial therapy for pneumonia in 17/21 (81%) patients (Supplementary Tables 3–4 in Supplement Materials). In the Per Protocol population, plasma mcfDNA sequencing identified ≥1 probable cause of a clinically relevant infection other than pneumonia in 67/173 (38.7%) patients (Supplementary Table 5 in Supplement Materials).

### Agreement

Among 52 patients in the Per Protocol population with a probable cause of pneumonia identified by usual care testing, plasma mcfDNA identified the same pathogen in 27 (positive percent agreement 52%, 95% CI, 38% to 66%) (Table 2). Of 121 patients with negative usual care testing, plasma mcfDNA did not identify a probable cause of pneumonia in 100 (negative percent agreement 83%, 95% CI, 75% to 89%). Positive percent agreement was highest when a DNA viral pathogen was identified as a probable cause of pneumonia by usual care testing (100%) and lowest when a fungal pathogen was identified (43%) (Supplementary Tables 6–8 in Supplement Materials). *Aspergillus* species were adjudicated as a causative pathogen in 14/15 (93%) patients with fungal pneumonia exclusively identified by usual care.

**Table 2.** **Measures of Agreement Between Usual Care Testing and Plasma Microbial Cell-Free DNA Sequencing (Per Protocol Population** ^a^**)**

		All Usual Care Testing		
		Positive	Negative	Total
Plasma mcfDNA sequencing^b^	Positive	27	21	48
	Negative	25^c^	100	125
	Total	52	121	173
Measure	Percent (95% confidence limits^d^)			
Positive percent agreement	51.9 (37.6, 66.0)			
Negative percent agreement	82.6 (74.7, 88.9)			

Abbreviations: DNA, deoxyribonucleic acid; mcfDNA, microbial cell-free DNA.

^a^The Per Protocol Population included patients with complete protocol-required testing, a valid plasma mcfDNA sequencing test collected within 24 hours of enrollment, and no protocol deviations.

^b^Plasma mcfDNA sequencing does not detect RNA viral pathogens.

^c^Includes 4 RNA viruses identified by usual care testing and adjudicated as probable cause of pneumonia.

^d^Clopper-Pearson confidence intervals.

## DISCUSSION

In this study, adding plasma mcfDNA sequencing to conventional diagnostic standards increased the absolute diagnostic yield by 12%. These findings suggest that one probable cause of pneumonia would be exclusively identified by plasma mcfDNA sequencing for approximately every 8 patients tested at the time of diagnostic bronchoscopy. Plasma mcfDNA sequencing identified a pathogenic cause of pneumonia in 17% of immunocompromised patients with no microbial diagnosis by usual care. Moreover, in most of these plasma mcfDNA sequencing-only diagnoses, the results were adjudicated as likely to lead to treatment changes.

Despite a thorough diagnostic evaluation, an adjudicated probable cause of pneumonia was identified by usual care testing in only 30% of enrolled patients. This proportion is lower than that reported in some previous studies; however, we believe the study design provides an accurate real-world estimate of diagnostic yield for pneumonia in this patient population [7]. Notably, we implemented a structured, centralized clinical adjudication process that likely resulted in a lower proportion of pneumonia cases with a confirmed microbiologic cause when compared to previous studies based on positive diagnostic tests alone. Acute noninfectious pulmonary conditions that complicate hematopoietic cell transplantation and treatment of hematologic malignancy were not adjudicated. Although all enrolled patients underwent bronchoscopy for clinically suspected pneumonia, it is likely some had a noninfectious pulmonary disease. Moreover, patients were enrolled irrespective of bronchoscopy timing relative to symptom onset or prior empiric antimicrobial exposure, 2 known contributors to the low yield of bronchoscopic testing in this patient population [2, 12, 13]. Because the usual care comparator included all testing collected in routine care to establish a pneumonia etiology, the results reflect the extent of diagnostic testing and the real-world variability of both local testing standards and diagnostic yield in these patients. Taken together, our study findings enhance understanding of contemporary diagnostic yield and underscore the need for improved diagnostic testing in severely immunocompromised patients undergoing bronchoscopy to establish an etiology of pneumonia.

Agreement between usual care and plasma mcfDNA sequencing varied by pathogen class and was lowest in patients with fungal pneumonia. *Aspergillus* species accounted for most fungal pneumonia pathogens identified exclusively by usual care. This finding is consistent with a prior retrospective study of plasma mcfDNA sequencing in invasive fungal disease [8]. Whether high rates of mold-active antifungal prophylaxis in enrolled patients reduced plasma mcfDNA sequencing yield is unknown. Fungal pneumonia diagnosis often relies on non-culture methods like galactomannan, which may lead to overdiagnosis or be differentially impacted by prior mold-active antifungal exposure [14]. In most patients where an *Aspergillus* species was adjudicated as the probable cause of pneumonia, mycological criteria were fulfilled by galactomannan testing alone. Although the mechanisms underlying discrepant diagnostic yield in these patients are unknown, plasma mcfDNA sequencing did identify invasive fungal infections not diagnosed by usual care. These findings highlight the complementary nature of a diagnostic testing approach that combines plasma mcfDNA sequencing with contemporary standards of care [8].

Strengths of this study include the prospective, multicenter design and blinded, multistage committee adjudication of all key study endpoints. Additionally, the study required a rigorous usual care evaluation to ensure a robust estimation of the additive diagnostic value of plasma mcfDNA sequencing. We conservatively defined additive diagnostic value to include only patients with a probable cause of pneumonia identified exclusively by plasma mcfDNA sequencing—against the background of a protocol-specified usual care testing battery. However, routine care diagnostic testing is often less extensive, and bronchoscopy may be delayed or avoided. Consequently, this study may underestimate the real-world additive diagnostic value of non-invasive plasma mcfDNA sequencing. Future studies should evaluate whether combining plasma mcfDNA sequencing with usual care testing may offer other forms of additive value by identifying clinically relevant coinfections and reducing time to pathogen identification, need for additional diagnostic testing, cost, and risk of procedural complications.

This study has limitations. First, approximately one-quarter of enrolled patients were excluded from the primary analysis because minimum diagnostic standard testing was incomplete. Most of these patients were excluded for missing blood cultures, a test with low diagnostic yield in immunocompromised patients with pneumonia [15, 16]. Because additive diagnostic value was similar in the Per Protocol population and among all enrolled patients with plasma mcfDNA sequencing, it is unlikely that excluding patients without blood cultures systematically biased study findings. Second, the impact of prior antimicrobial exposure on plasma mcfDNA sequencing yield could not be assessed as most patients were receiving empiric broad antibacterial therapy and mold-active antifungal therapy upon enrollment. Finally, the study did not require *Aspergillus* serum or bronchoalveolar lavage fluid PCR testing, which has been recommended by clinical practice guidelines for severely immunocompromised patients with suspected invasive pulmonary aspergillosis [17].

In conclusion, non-invasive plasma microbial cell-free DNA sequencing increased detection of the microbial etiology of pneumonia in severely immunocompromised patients undergoing a rigorous invasive diagnostic evaluation. Collectively, these data support adoption of plasma mcfDNA sequencing to improve diagnostic yield in this high-risk population.

## Supplementary Data

Supplementary materials are available at *Clinical Infectious Diseases* online. Consisting of data provided by the authors to benefit the reader, the posted materials are not copyedited and are the sole responsibility of the authors, so questions or comments should be addressed to the corresponding author.

## Supplementary Material

ciad599_Supplementary_Data

## Notes

***Author Contributions*.** All authors reviewed and approved the final version of this manuscript before submission. S. P. B.: Conceptualization, enrollment, data interpretation, writing (first draft), review, and editing. R. F. C.: Enrollment, writing, review, and editing. S. D.: Enrollment, writing, review, and editing. J. A. H.: Enrollment, writing, review, and editing. Y. J. L.: Enrollment, writing, review, and editing. G. H.: Enrollment, writing, review, and editing. A. L.: Enrollment, writing, review, and editing. A. D.: Enrollment, writing, review, and editing. P. V, C.-H.: Enrollment, writing, review, and editing. E. B.: Enrollment, writing, review, and editing. F. K.: Enrollment, writing, review, and editing. D. N.: Enrollment, writing, review, and editing. G. A. P.: Enrollment, writing, review, and editing. C. B. S.: Enrollment, writing, review, and editing. M. F.: Enrollment, writing, review, and editing. M. A. B.: Enrollment, writing, review, and editing. T. D.: Writing, review, and editing. M. T. M.: Conceptualization, writing, review, and editing. E. K. M.: Writing, review, and editing. D. B, M.: Writing, review, and editing. A. D. B.: Writing, review, and editing. D. L. G.: Writing, review, and editing. J. L. T.: Writing, review, and editing. C. E. B.: Writing, review, and editing. R. B.: Conceptualization, methodology, analysis, writing, review, and editing. J. D. L.: Methodology, analysis, writing, review, and editing. E. L. T.: Conceptualization, writing, review, and editing. O. W.: Writing, review, and editing. M. M.: Analysis, writing, review, and editing. D. H.: Conceptualization, writing, review, and editing. R. D.: Methodology, writing, review, and editing. D. S, L.: Data interpretation, writing, review, and editing. S. B.: Writing, review, and editing. B. A. P,: Data interpretation, writing, review, and editing. T. A. B.: Conceptualization, data interpretation, writing, review, and editing. V. G. F.: Conceptualization, data interpretation, writing, review, and editing. T. L. H.: Conceptualization, data interpretation, writing (first draft), review, and editing.

***Acknowledgments*.** The authors thank the patients and families whose willingness to contribute to science made this research study possible. They also thank Constance Lau for providing data validation support and biostatistical consultation as well as Ravi Tamiri for support with data cleaning, structuring, and creation of the interactive online dashboard of study results.

***Financial support.*** This work was supported by Karius Inc, Redwood City, California, USA. Investigators from Karius were directly involved in design and conduct of the study; collection, management, analysis, and interpretation of the data; preparation, review, and approval of the manuscript; and decision to submit the manuscript for publication. Thomas E Davis reports support for this work in the form of testing performed at IU Health Pathology Lab and Pathology Research Lab: Galactomanan; BioFire RVP; Diasorin PJP PCR; Fungal culture and stain; respiratory culture and stain; AFB culture and stain (IU Health Pathology Lab and IU Pathology Research Lab were compensated as budgeted at lab standard rates for tests performed and listed in the protocol and contract). J. D. L. reports no support outside of his salary working as a statistician for the Duke Clinical Research Institute. Y. J. L. reports receiving institutional research support for clinical trial from Karius and support for this work form Scynexis.
