# Supplementary material for: Plasma Microbial Cell-Free DNA Sequencing in Immunocompromised Patients With Pneumonia: A Prospective Observational Study
Source: Clin Infect Dis. 2023 Oct 10;78(3):775–84. doi: 10.1093/cid/ciad599 (PMC10954333; doi:10.1093/cid/ciad599)
Supplement: ciad599_Supplementary_Data [file ciad599_supplementary_data.docx]

**Supplemental Online Content**

**Plasma Microbial Cell-Free DNA Sequencing in Immunocompromised Patients with Pneumonia: A Prospective Observational Study**

This appendix has been provided by the authors to give readers additional information about the work.

Table of Contents

[Section 1: Methods 3](#_Toc134201735)

[Plasma Microbial Cell-Free DNA Sequencing Test Procedure 3](#_Toc134201736)

[Example Plasma Microbial Cell-Free DNA Sequencing Test Report 4](#_Toc134201737)

[Study Definitions 8](#_Toc134201738)

[Study Analysis Populations 10](#_Toc134201739)

[Clinical Events Classification (CEC) Adjudication Process 11](#_Toc134201740)

[eFigure. Minimum Diagnostic Standard 13](#_Toc134201741)

[Section 2: Results 14](#_Toc134201742)

[Interactive Dashboard of PICKUP Study Results 14](#_Toc134201743)

[eTable 1. Diagnostic Testing and Clinical Adjudication Results for Patients with an Identified Pneumonia Etiology 16](#_Toc134201744)

[eTable 2. Diagnostic Testing Results for Patients with Discordant Fungal Pneumonia Diagnoses 29](#_Toc134201745)

[eTable 3. Adjudicated Possible Changes in Antimicrobial Therapy Among Patients with a Pneumonia Etiology Exclusively Identified by Plasma mcfDNA Sequencing 32](#_Toc134201746)

[eTable 4. Usual Care Testing, Potential Antimicrobial Changes, and Mortality in Patients with a Pneumonia Etiology Exclusively Identified by Plasma Microbial Cell-Free DNA Sequencing 34](#_Toc134201747)

[eTable 5. Clinically Relevant Non-Pneumonia Infections Identified by Plasma Microbial Cell-Free DNA Sequencing 37](#_Toc134201748)

[eTable 6. Measures of Agreement Between Usual Care Testing and Plasma Microbial Cell-Free DNA Sequencing – Fungal Etiologies 38](#_Toc134201749)

[eTable 7. Measures of Agreement Between Usual Care Testing and Plasma Microbial Cell-Free DNA Sequencing – Bacterial Etiologies 39](#_Toc134201750)

[eTable 8. Measures of Agreement Between Usual Care Testing and Plasma Microbial Cell-Free DNA Sequencing – Viral Etiologies 40](#_Toc134201751)

Section 1: Methods

Plasma Microbial Cell-Free DNA Sequencing Test Procedure

The Karius Test® was developed and validated in the Karius Clinical Laboratory Improvements Amendments-certified/College of American Pathologists-accredited/New York State Department of Health-approved laboratory (Redwood City, California) to detect and quantify mcfDNA in plasma. After collection, blood samples were processed, and plasma separated and shipped to Karius following the Karius Specimen Collection & Preparation Instructions protocol (<https://kariusdx.com/karius-test/karius-test-process#specimen-collection>). Following mcfDNA extraction, enriched library preparation, sequencing (Illumina NextSeq550), discarding of human sequences, and alignment of the remaining sequences to a curated database of reference genomic sequences, test results were generated using the Karius version 3.7 analytical pipeline. This bioinformatic pipeline was designed to detect and quantify 1,563 microbes across bacteria, DNA viruses, fungi, and other eukaryotes. Plasma mcfDNA of microorganisms that are determined to be significantly higher than real-time background control specimens were reported and quantified in molecules per microliter, which is equivalent to the number of sequencing reads per microliter of plasma.

Example Plasma Microbial Cell-Free DNA Sequencing Test Report


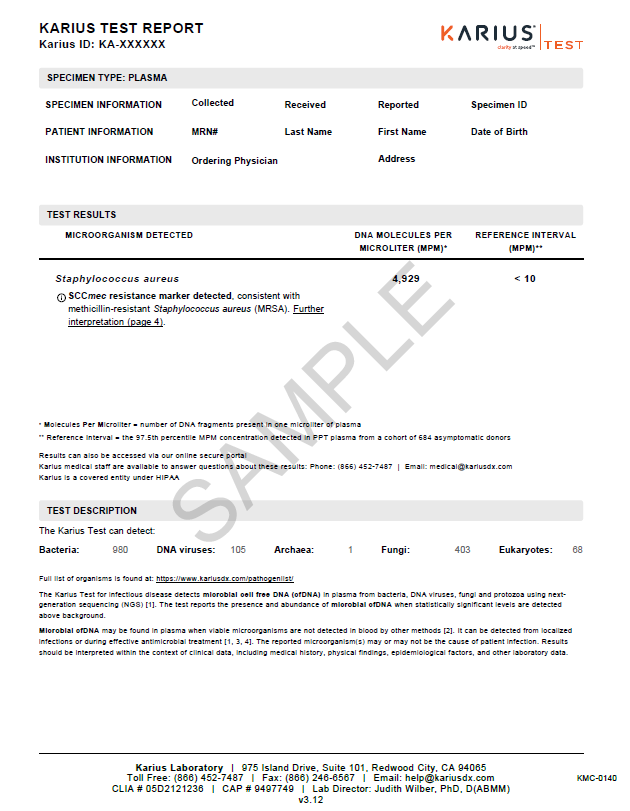


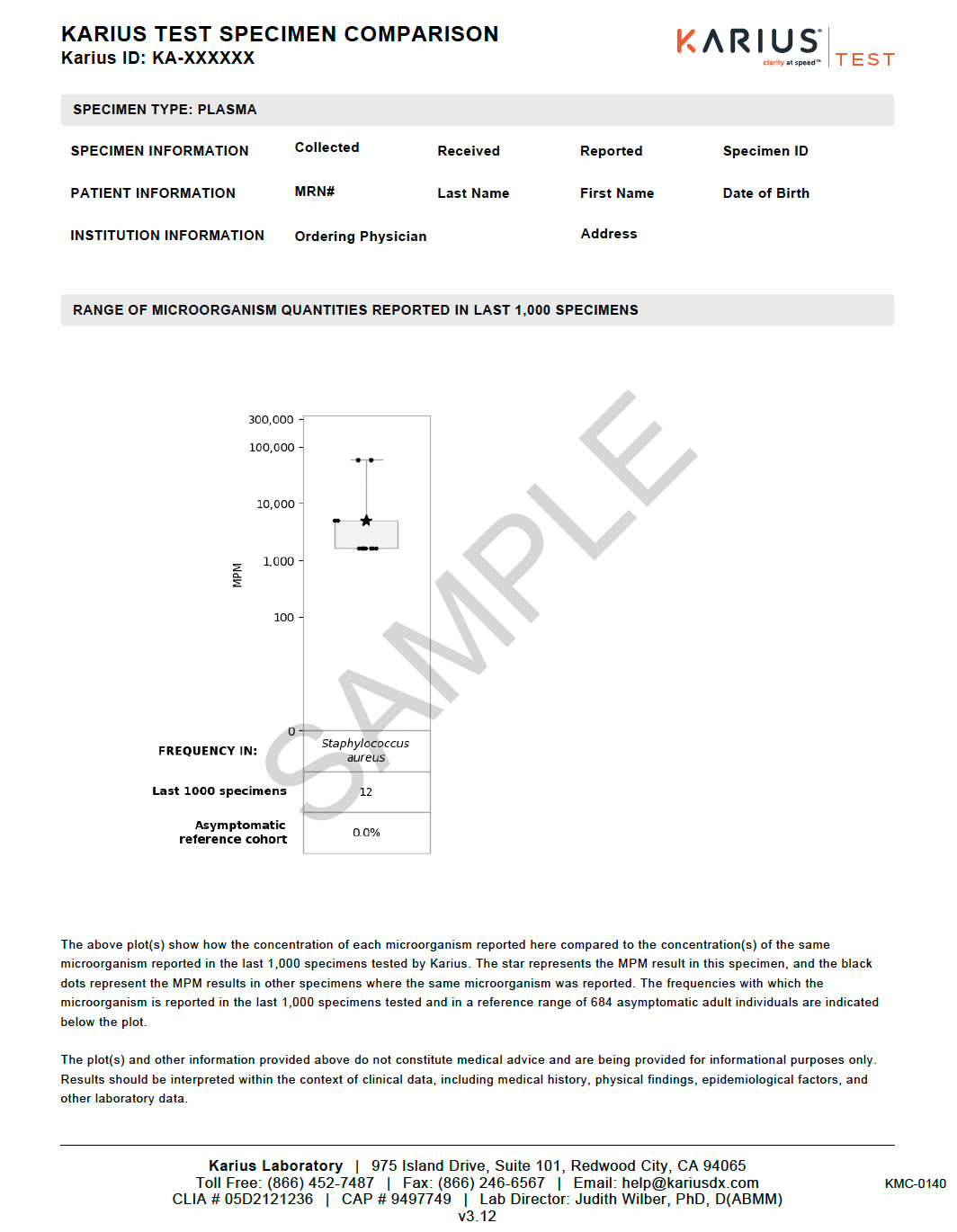


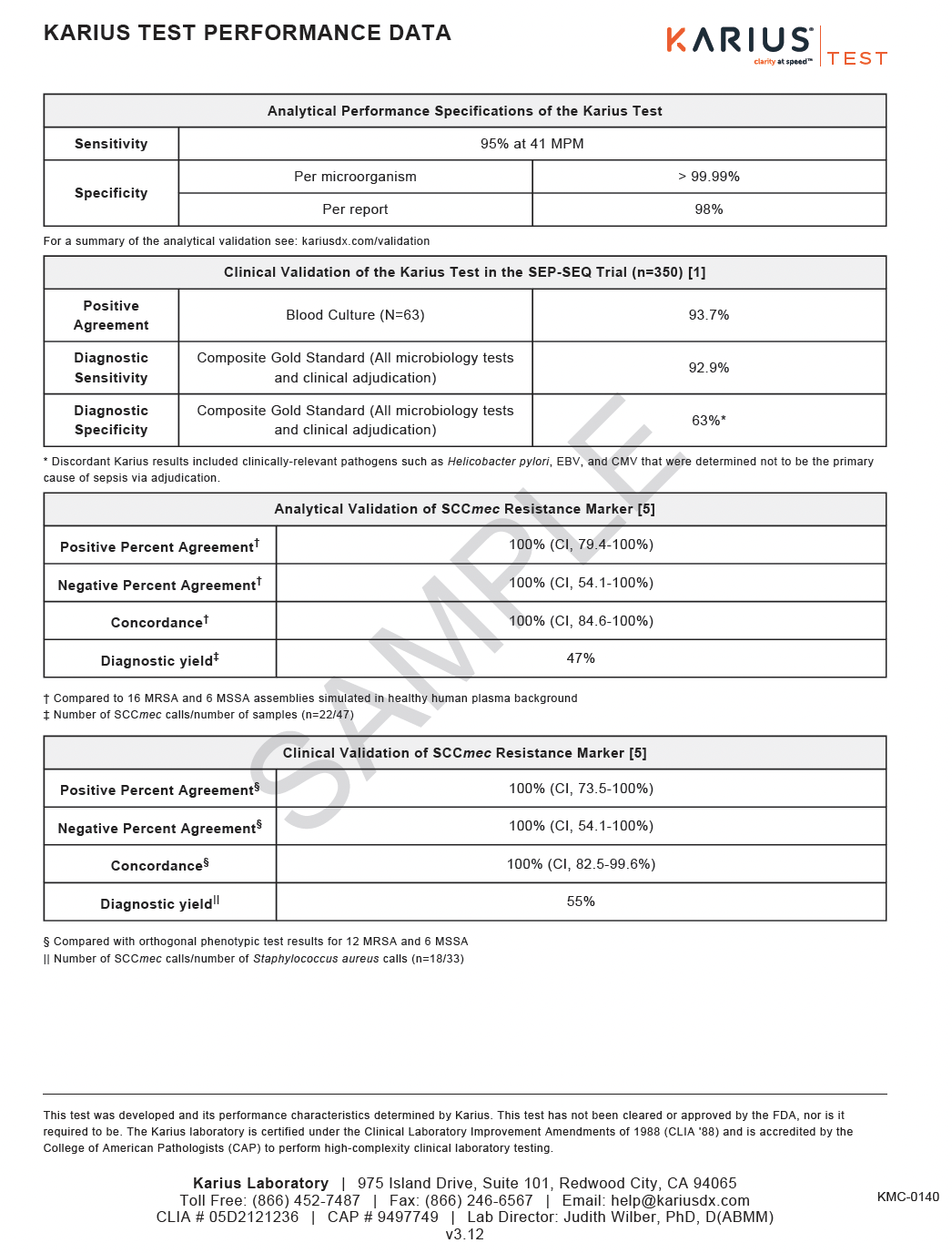


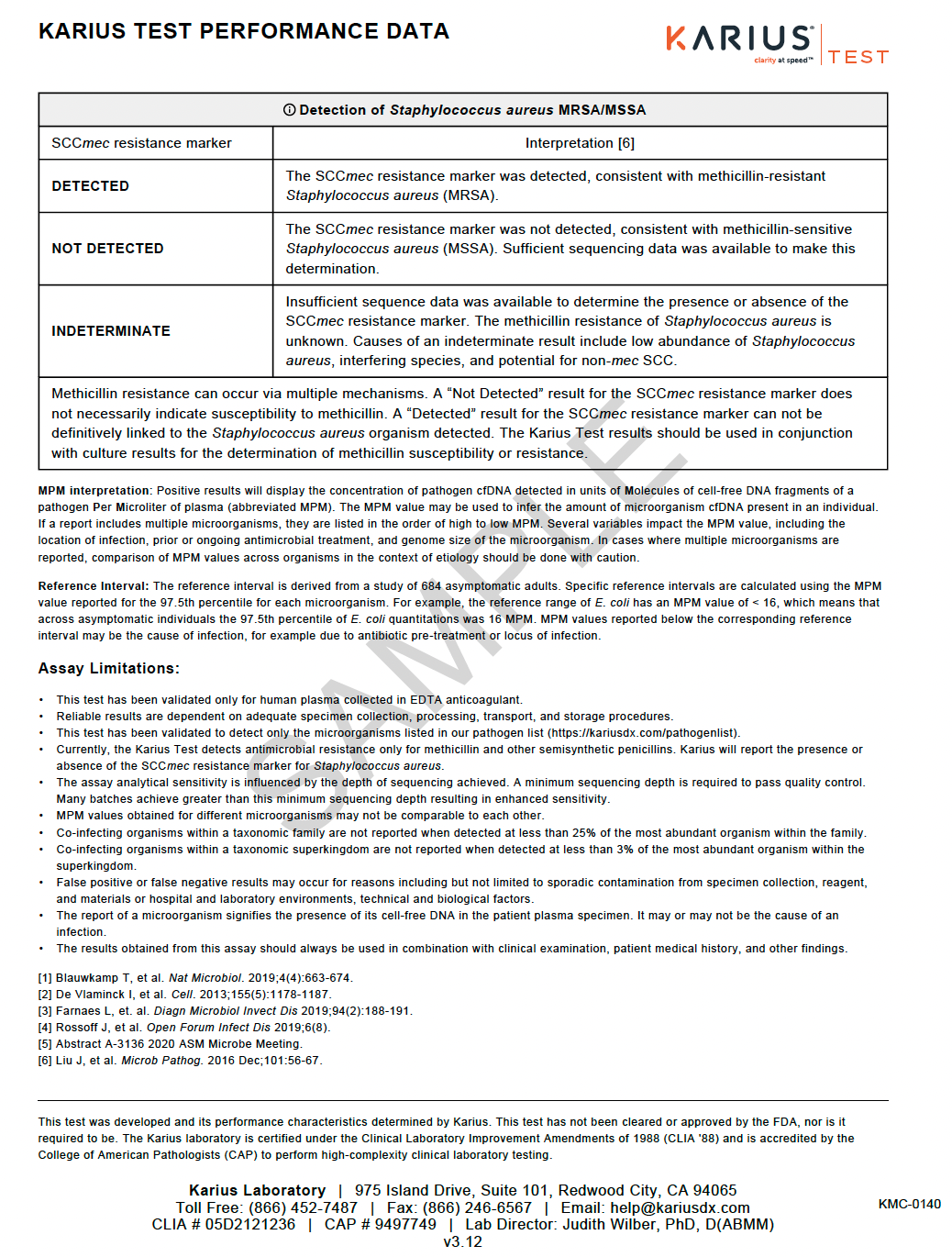


Study Definitions

Positive Agreement: Identification of the same microbe genus by a composite of all usual care testing and plasma microbial cell-free DNA sequencing. Only microbes adjudicated as a probable cause of pneumonia were included in the agreement analyses reported. Any other microbe(s) identified by either usual care or plasma microbial cell-free DNA sequencing that were not adjudicated as a cause of the patient’s index pneumonia event were excluded from all agreement analyses. The adjudicated composite of all usual care testing was considered the reference standard for defining true positive and negative diagnostic outcomes.

When usual care testing identified a probable cause of pneumonia (Microbe A) and plasma microbial cell-free DNA sequencing identified a different adjudicated probable cause of pneumonia (Microbe B), this event was classified as usual care positive, plasma microbial cell-free DNA negative (disagreement) irrespective of whether plasma microbial cell-free DNA sequencing identified a more likely primary cause of the index pneumonia event.

RNA viruses, which cannot be detected by plasma microbial cell-free DNA sequencing, adjudicated as a probable cause of pneumonia identified by usual care were retained in all agreement analyses. Consequently, if an RNA virus was adjudicated as the only probable cause of pneumonia, positive agreement was not possible.

Negative Agreement: No probable cause of pneumonia identified by either usual care testing or plasma microbial cell-free DNA sequencing.

Clinically relevant infections other than pneumonia (non-pneumonia infections): A microbe identified by plasma microbial cell-free DNA sequencing that was adjudicated as both 1) not a cause of the patient’s index pneumonia and 2) a probable cause of an active infection at another site. To best characterize clinical utility of plasma microbial cell-free DNA sequencing in this patient population, all microbes listed on the plasma microbial cell-free DNA sequencing report were classified by the Clinical Events Committee as either 1) a probable cause of the index pneumonia event, 2) a probable cause of a clinically relevant infection other than pneumonia, or 3) not causing an active infection (commensal organism or contaminant).

Usual care testing for non-pneumonia infections was not standardized or adjudicated by the Clinical Events Committee. Therefore, no comparisons between usual care testing and plasma microbial cell-free DNA sequencing for non-pneumonia infections are presented. Clinically relevant non-pneumonia infections identified by plasma microbial cell-free DNA sequencing are provided for descriptive purposes and to illustrate the potential clinical utility of this diagnostic platform in immunocompromised patients at risk for simultaneous pneumonia and non-pneumonia infections.

Study Analysis Populations

**Full analysis population:** all enrolled patients who met all study eligibility criteria

**Intent-to-diagnose population:** all eligible enrolled patients with at least 1 plasma microbial cell-free DNA sequencing test with valid result (defined as a specimen processed per protocol and passing internal quality control assessments). Plasma for microbial cell-free DNA sequencing was collected within 1 day of enrollment and on study days 3 and 5. Patients were included in the intent-to-diagnose population if a valid plasma microbial cell-free DNA sequencing test result was available from any sample collected. Only valid plasma microbial cell-free DNA sequencing test results from the sample collected within 1 day of enrollment (enrollment sample) were adjudicated by the Clinical Events Committee. Usual care testing results from all patients in the intent-to-diagnose population were adjudicated, irrespective of whether a valid enrollment plasma microbial cell-free DNA sequencing test result was available.

**Per Protocol population:** all patients with complete minimum diagnostic standard testing, a valid plasma mcfDNA sequencing test collected within 24 hours of enrollment, and no protocol deviations that might bias a comparison of usual care to plasma mcfDNA sequencing test results.

Clinical Events Classification (CEC) Adjudication Process

*Process*

Clinical data for each enrolled patient were reviewed independently by one Infectious Diseases and one Pulmonary physician. These adjudicators were blinded to the plasma mcfDNA result until they had assessed whether usual care testing had identified the probable cause of pneumonia. Once they had adjudicated usual care testing, they received the plasma mcfDNA testing result and adjudicated the clinical significance of any microbes listed in the mcfDNA sequencing report. Any disagreements between the two primary adjudicators were resolved by a committee discussion that included at least 3 additional physicians, at least one of whom was an Infectious Diseases and one a Pulmonary specialist. The CEC chair (TH) was present for all committee discussions to enhance consistency of adjudications. If the disagreement related to the usual care results, then the additional committee members were blinded to the plasma mcfDNA results.

*Approach to commensal flora*

Some organisms can be non-pathogenic normal flora of the gastrointestinal or respiratory tracts (commensals) but can also cause clinical disease, particularly in immunocompromised patients. Adjudicators used clinical information including host immune status, imaging results, bronchoscopy findings, and response to prior and subsequent antibiotic therapy to determine whether such organisms were probable causes of the patient’s pneumonia. For example, a patient with dysphagia with an indolent presentation and chest imaging showing involvement of the dependent portions of the lungs might be thought to have an aspiration pneumonia, and a plasma mcfDNA result with high level of molecules per microliter (MPM) of an oral anaerobe would be consistent with that clinical syndrome. In contrast, low MPM of oral flora would likely not be considered significant in a patient whose clinical syndrome was more suggestive of invasive pulmonary aspergillosis.

Despite inherent subjectivity in these clinical judgments, the multiple independent layers of multidisciplinary adjudication by physicians experienced in the care of these patients was felt to be the most rigorous available standard. Additionally, to support a quality control analysis, 26 (10%) cases were randomly selected and re-adjudicated by committee members who were blinded to and without knowledge of the original adjudication results. This re-adjudication did not result in any changes to the initial results.

eFigure. Minimum Diagnostic Standard


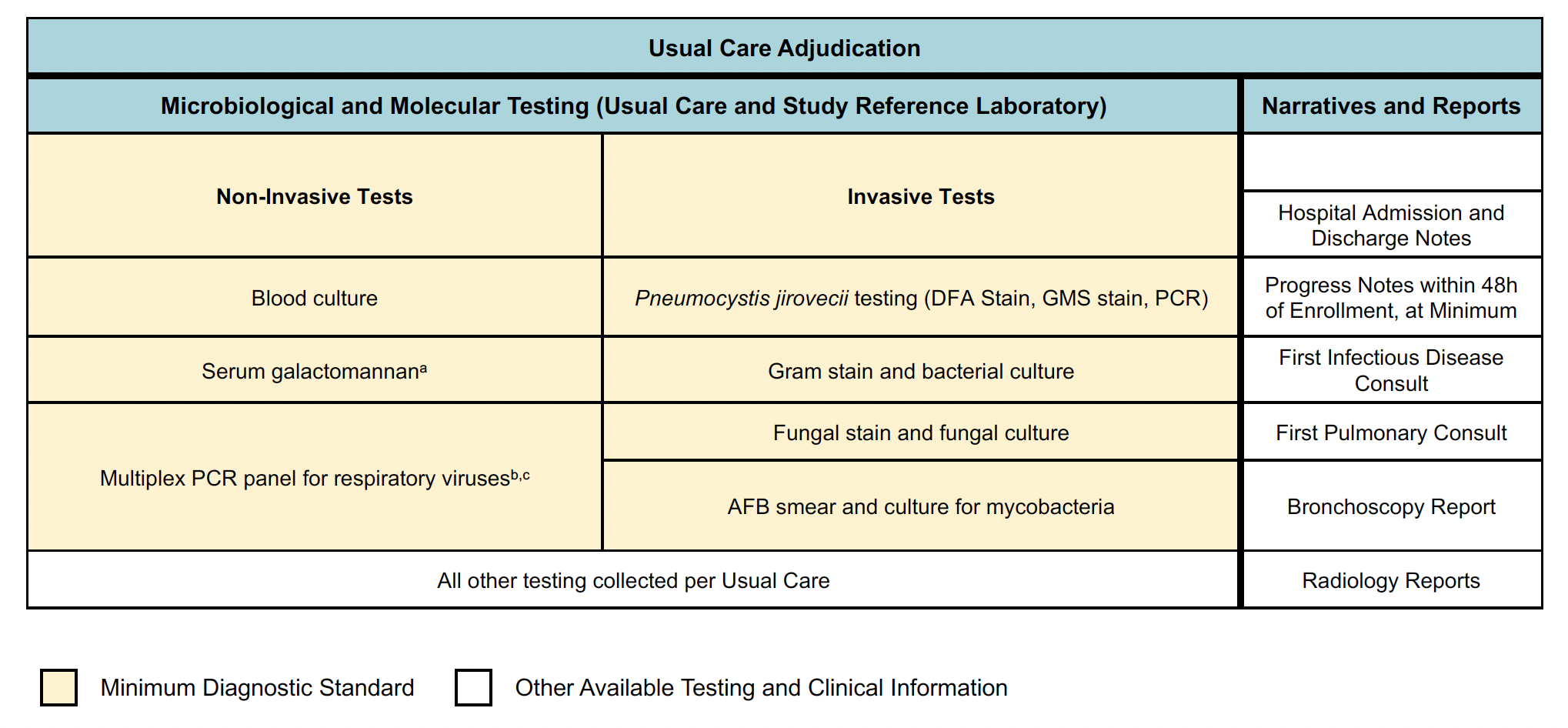


^a^ Bronchoalveolar lavage fluid galactomannan testing could fulfill the minimum diagnostic standard testing requirement when a serum galactomannan test was not collected.

^b^ Multiplex PCR testing for respiratory viruses from a nasopharyngeal wash or bronchoalveolar lavage fluid sample collected in usual care could fulfill the minimum diagnostic standard testing requirement when a study nasopharyngeal swab sample was not collected.

^c^ The minimum diagnostic standard required multiplex PCR testing for the following respiratory viruses: Influenza A and B, Parainfluenza 1, 2, 3, and 4, Adenovirus, Respiratory syncytial virus, Human metapneumovirus, and Enterovirus/Rhinovirus.

Abbreviations: AFB, acid fast bacilli; DFA, direct fluorescent antibody; GMS, Grocott methenamine silver; PCR, polymerase chain reaction.

Section 2: Results

**Pneumonia Diagnostic Testing and Outcomes**

Interactive Dashboard of PICKUP Study Results

A publicly available dashboard for the PICKUP study is available at: <http://bit.ly/3E7fjRx>

This dashboard includes all the raw data collected in electronic case report forms, Clinical Events Committee adjudication, and plasma mcfDNA sequencing results (abbreviated as KT for Karius Test) for the Per Protocol population. Some fields have been redacted to protect personal health information. The dashboard allows users to explore the extent of diagnostic testing and clinical data reviewed by the Clinical Events Committee and understand the complexity of usual care testing in greater detail than can be provided in the manuscript.

**eTable 1**. This table summarizes usual care diagnostic testing, plasma microbial cell-free DNA sequencing, and clinical adjudication results for patients in the Per Protocol population who had an etiology of pneumonia identified. This table is also available in an online version at: <https://kariusdx.com/pickup-study/pickup-study-manuscript-table>

**eTable 2.** This table summarizes usual care testing, microbial cell-free DNA sequencing, and post-hoc *Aspergillus* PCR results for patients with molds adjudicated as a probable cause of pneumonia exclusively by usual care testing or microbial cell-free DNA sequencing. Post-hoc *Aspergillus* PCR testing was not performed in patients with concordant usual care and microbial cell-free DNA sequencing (both identified the same mold genus as a probable cause of pneumonia).

**Potential Clinical Utility Assessments**

**eTable 3.** This table summarizes potential changes in antimicrobial therapy had plasma microbial cell-free DNA sequencing test results been available in real time. Potential changes were adjudicated by the Clinical Events Committee. Results are presented for patients with a probable cause of pneumonia exclusively identified by plasma microbial cell-free DNA sequencing.

**eTable 4.** This table summarizes usual care testing performed that could have potentially identified the adjudicated cause of pneumonia, adjudicated potential changes in antimicrobial therapy had plasma microbial cell-free DNA sequencing results been clinically available in real time, and 30-day mortality in patients with an etiology of pneumonia exclusively identified by plasma microbial cell-free DNA sequencing.

**eTable 5.** This table summarizes the types of adjudicated clinically relevant infections other than pneumonia identified by plasma microbial cell-free DNA sequencing.

**Measures of Agreement**

**eTable 6.** This table summarizes agreement between a composite of all usual care testing and plasma microbial cell-free DNA sequencing for fungal etiologies of pneumonia.

**eTable 7.** This table summarizes agreement between a composite of all usual care testing and plasma microbial cell-free DNA sequencing for bacterial etiologies of pneumonia.

**eTable 8.** This table summarizes agreement between a composite of all usual care testing and plasma microbial cell-free DNA sequencing for viral etiologies of pneumonia


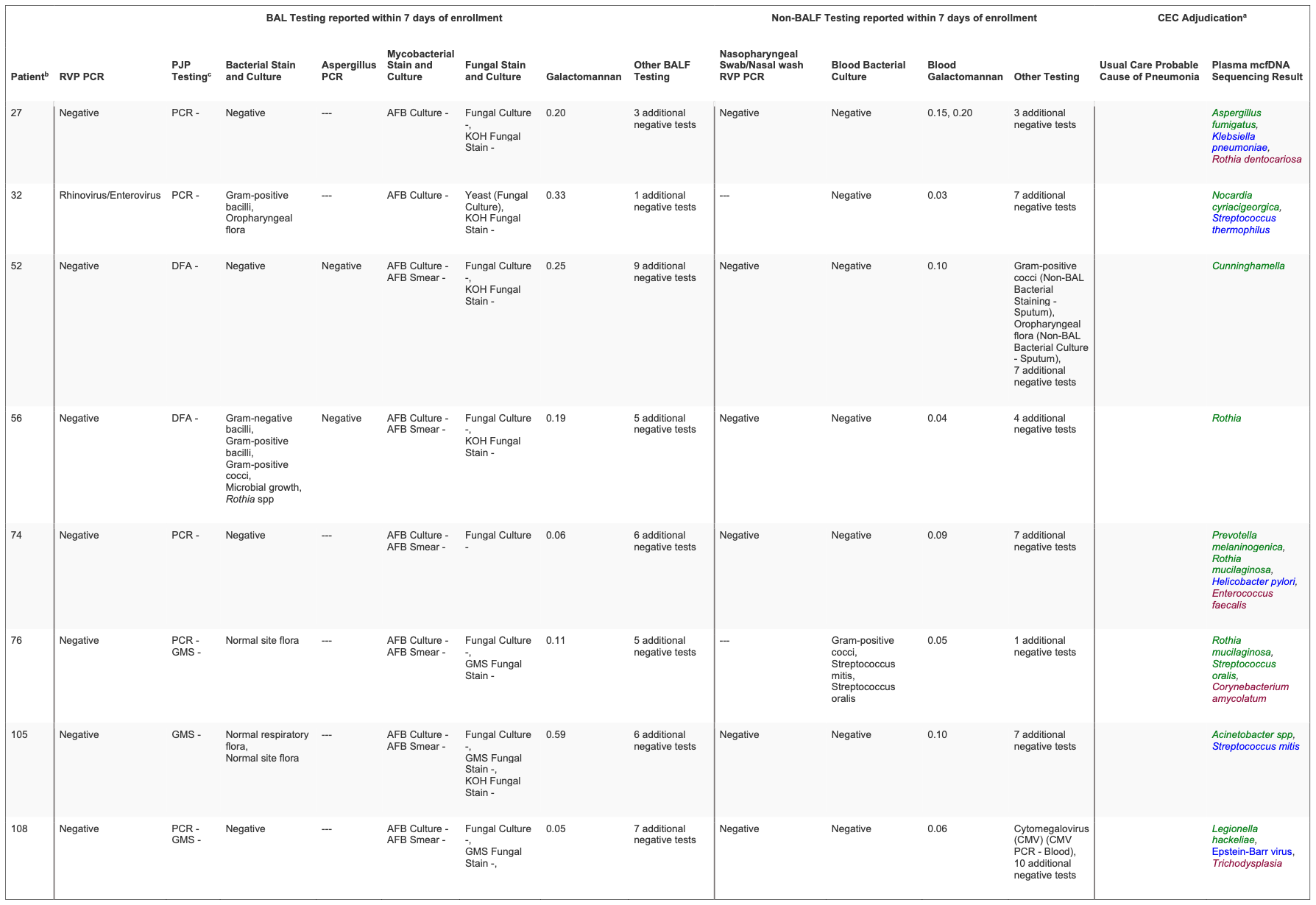
eTable 1. Diagnostic Testing and Clinical Adjudication Results for Patients with an Identified Pneumonia Etiology.


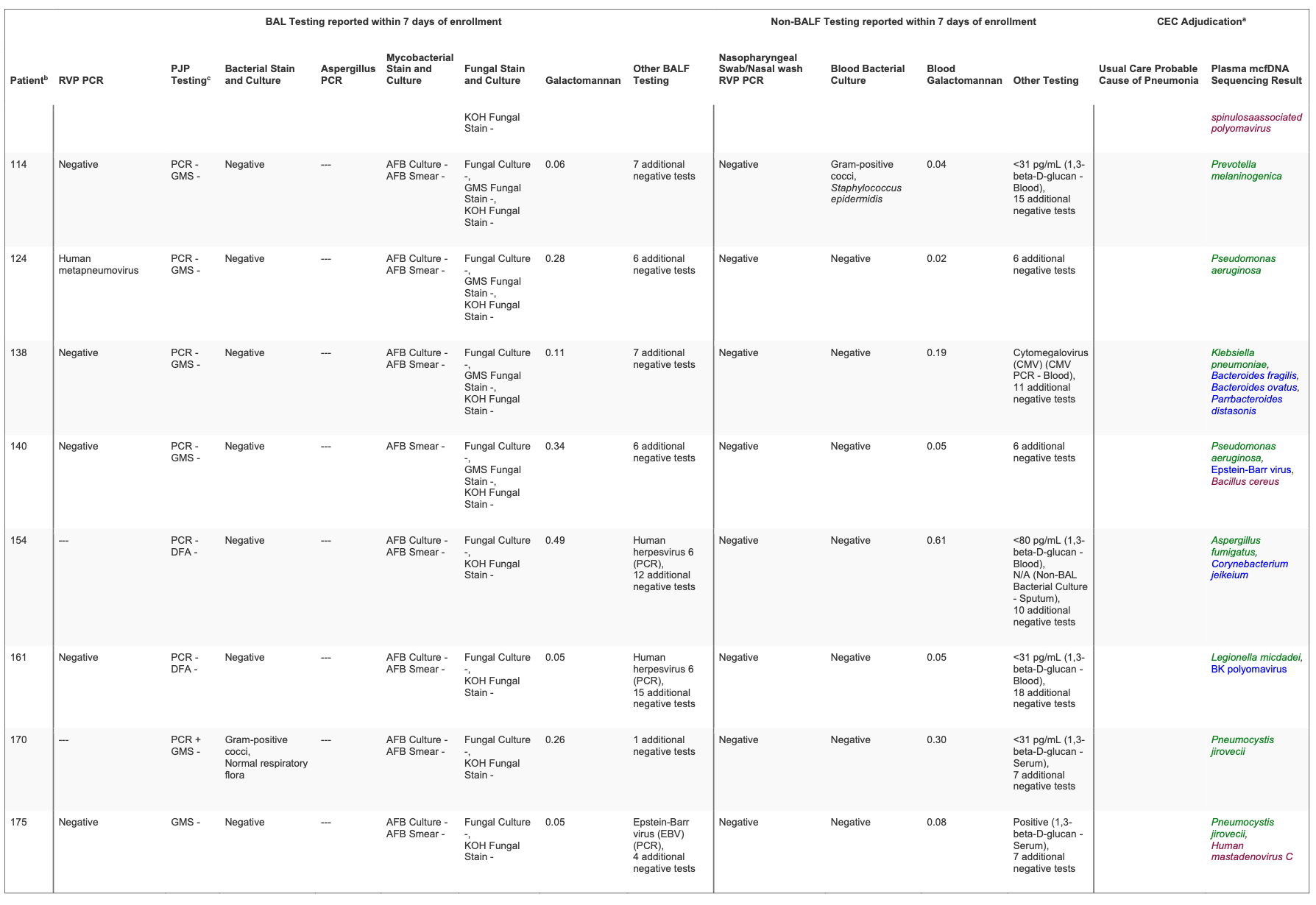


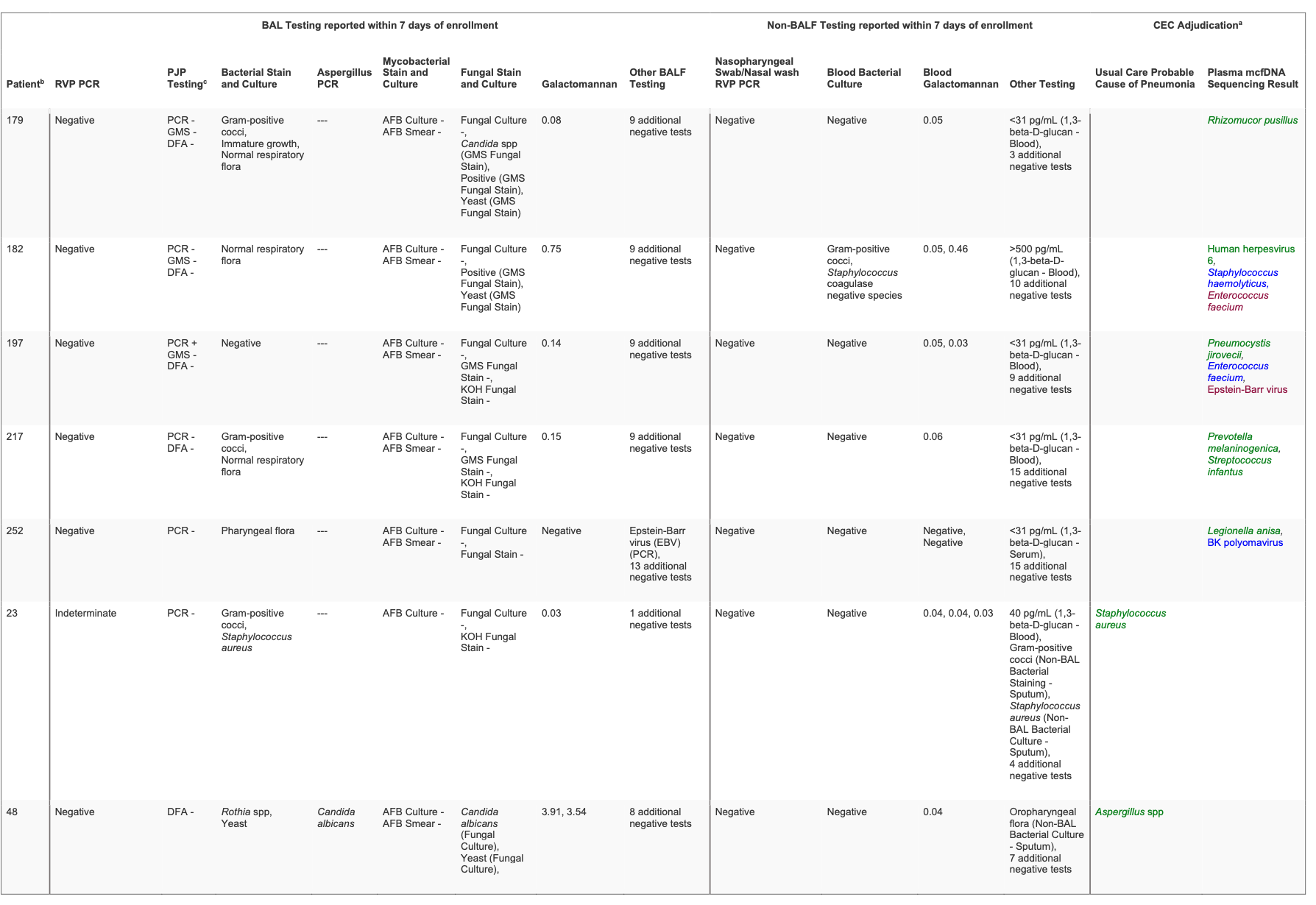


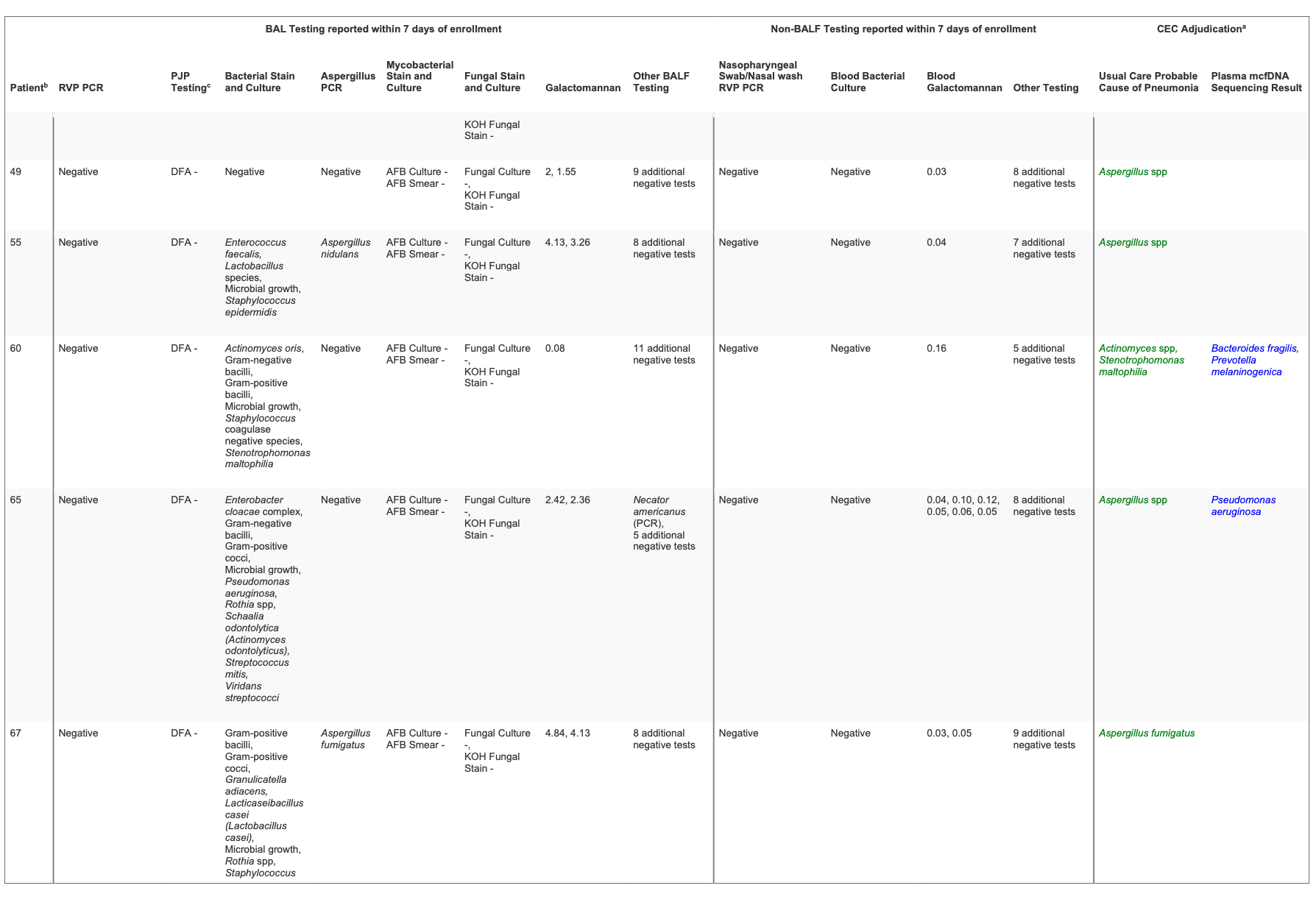


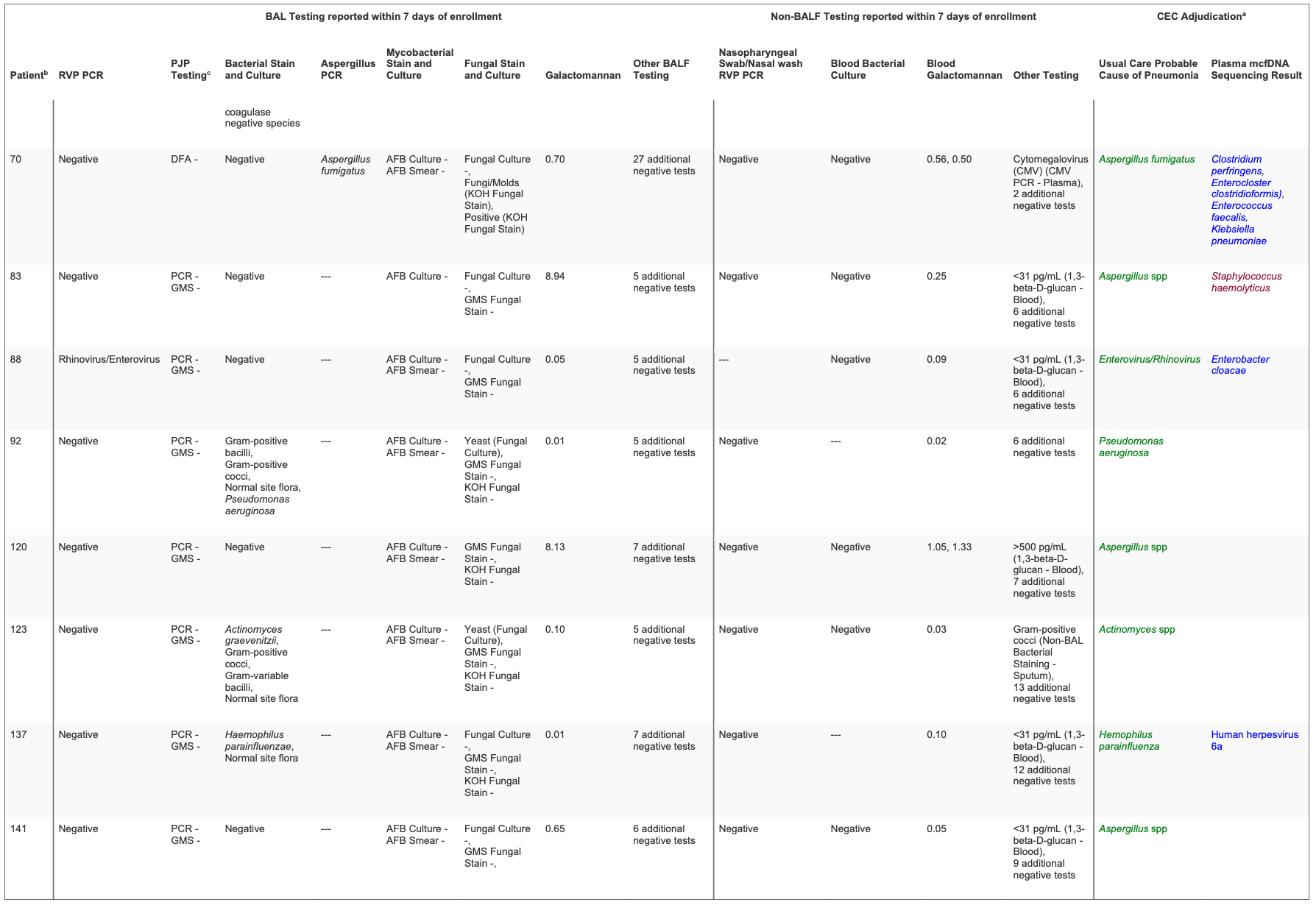


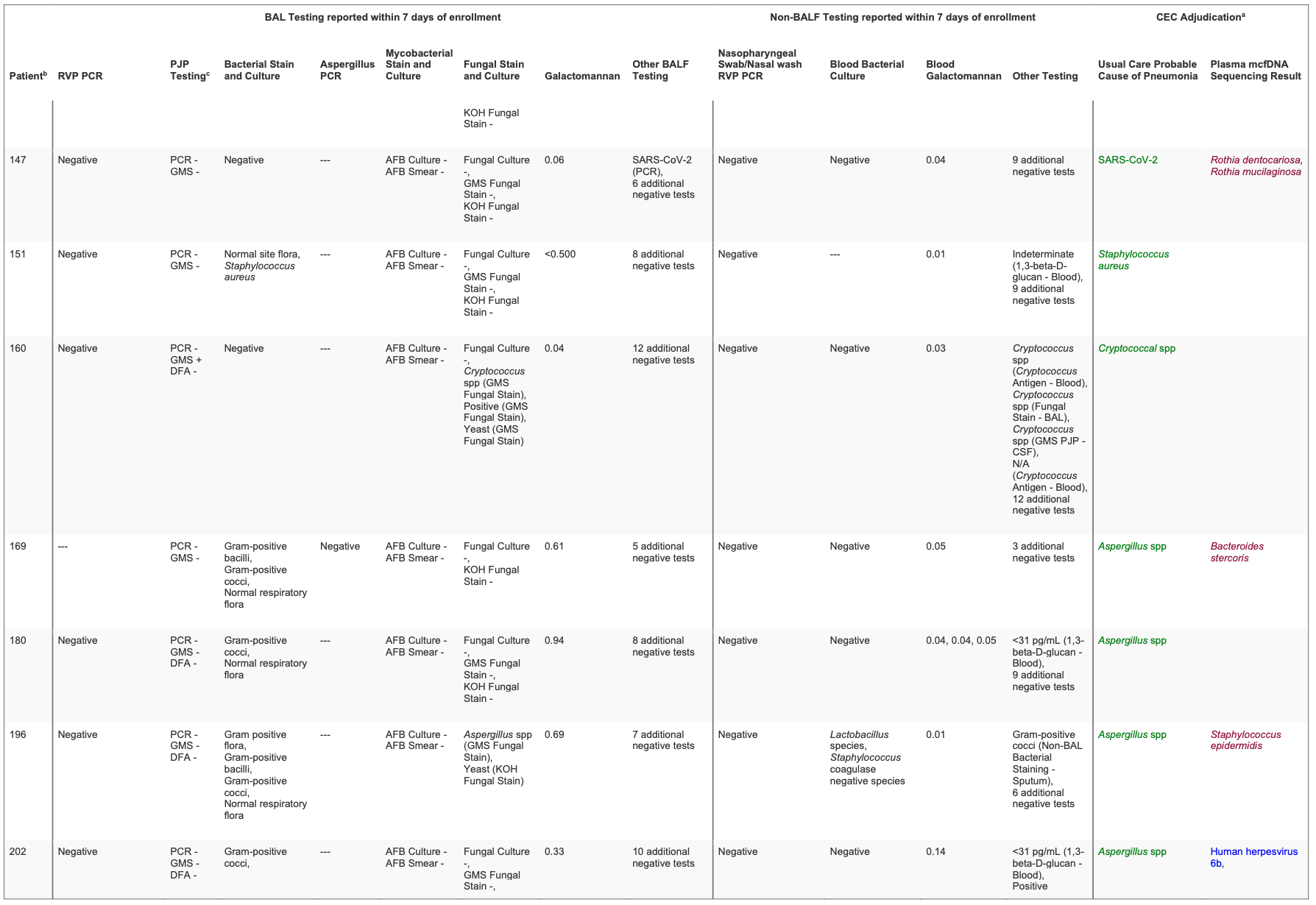


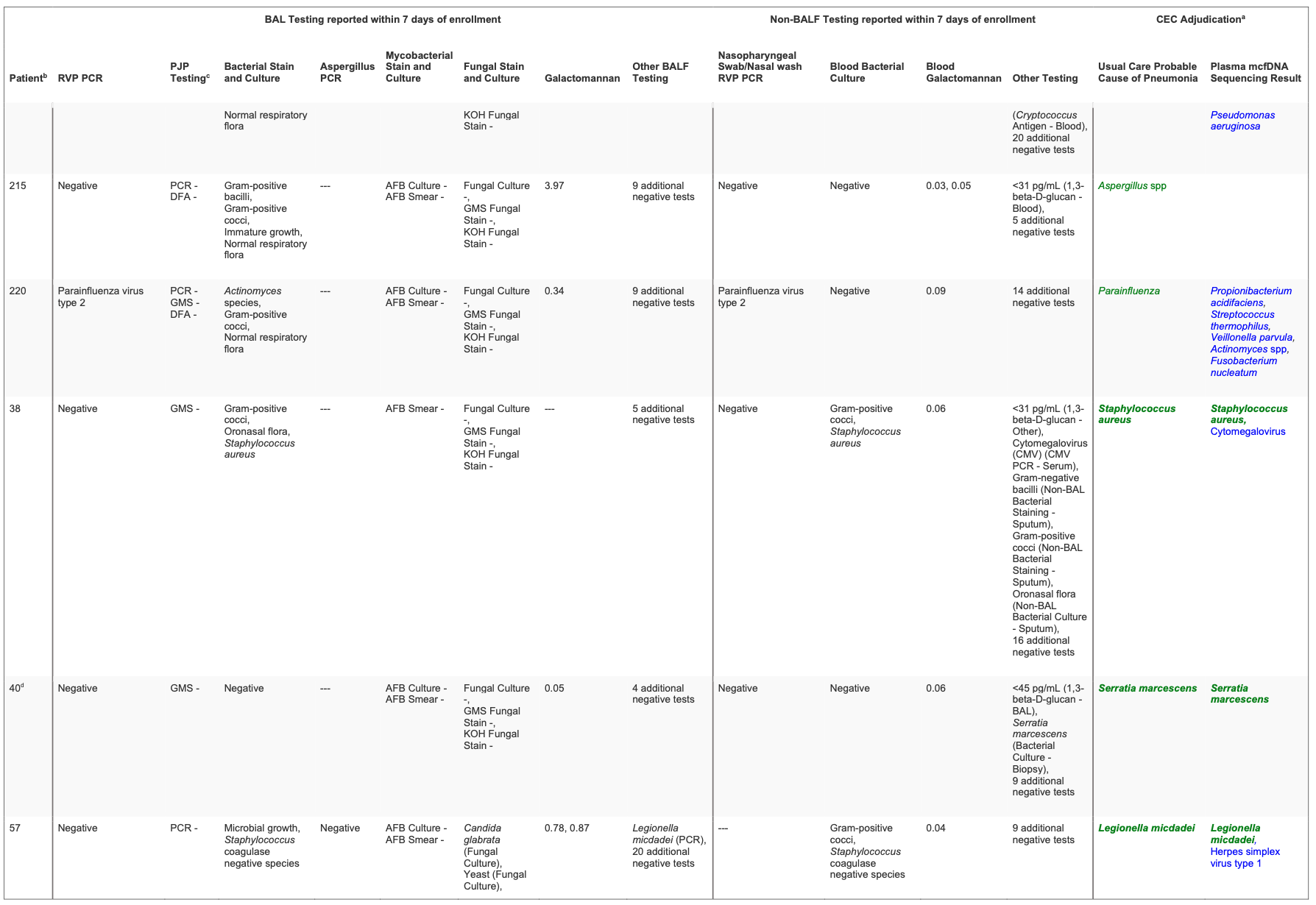


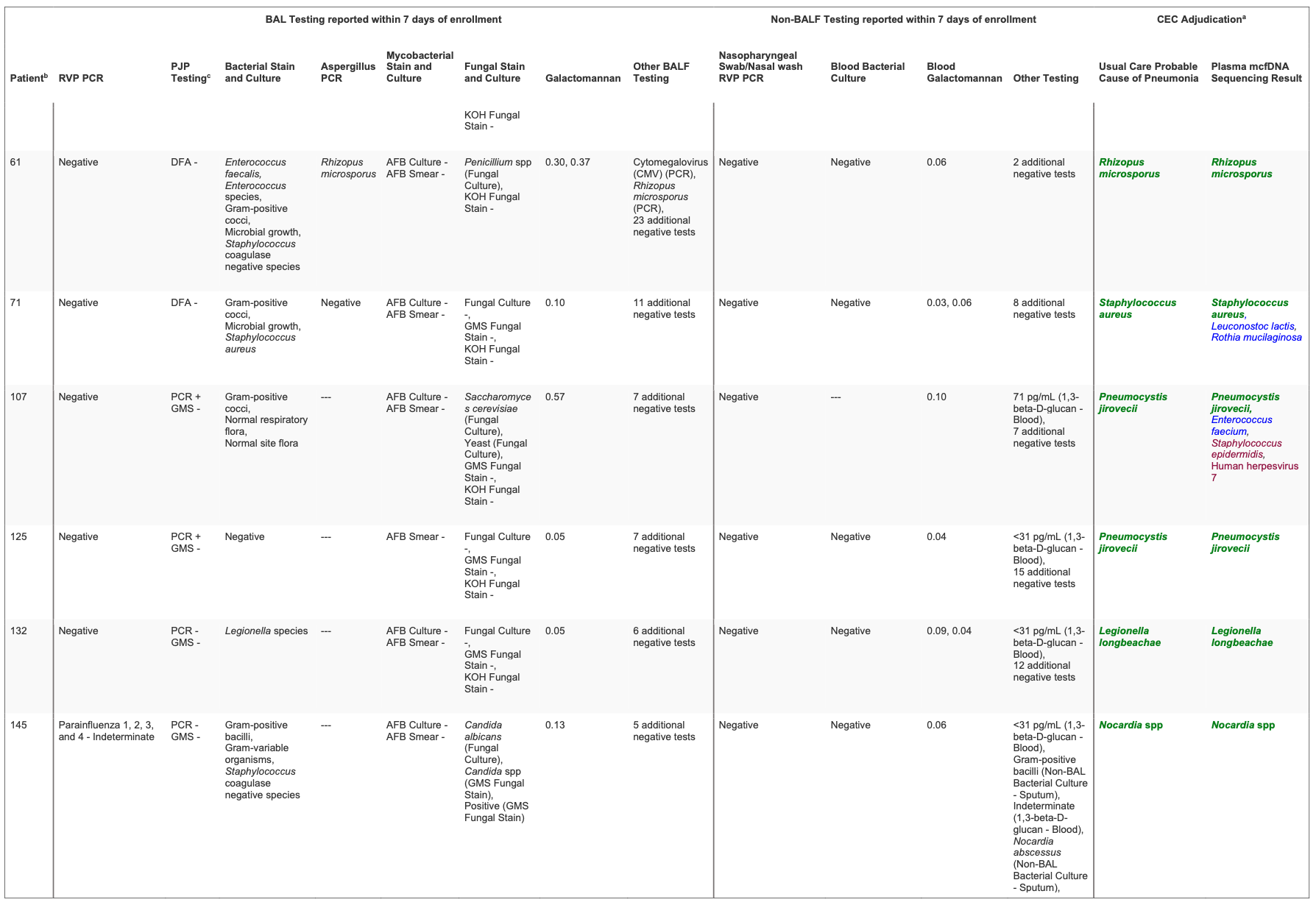


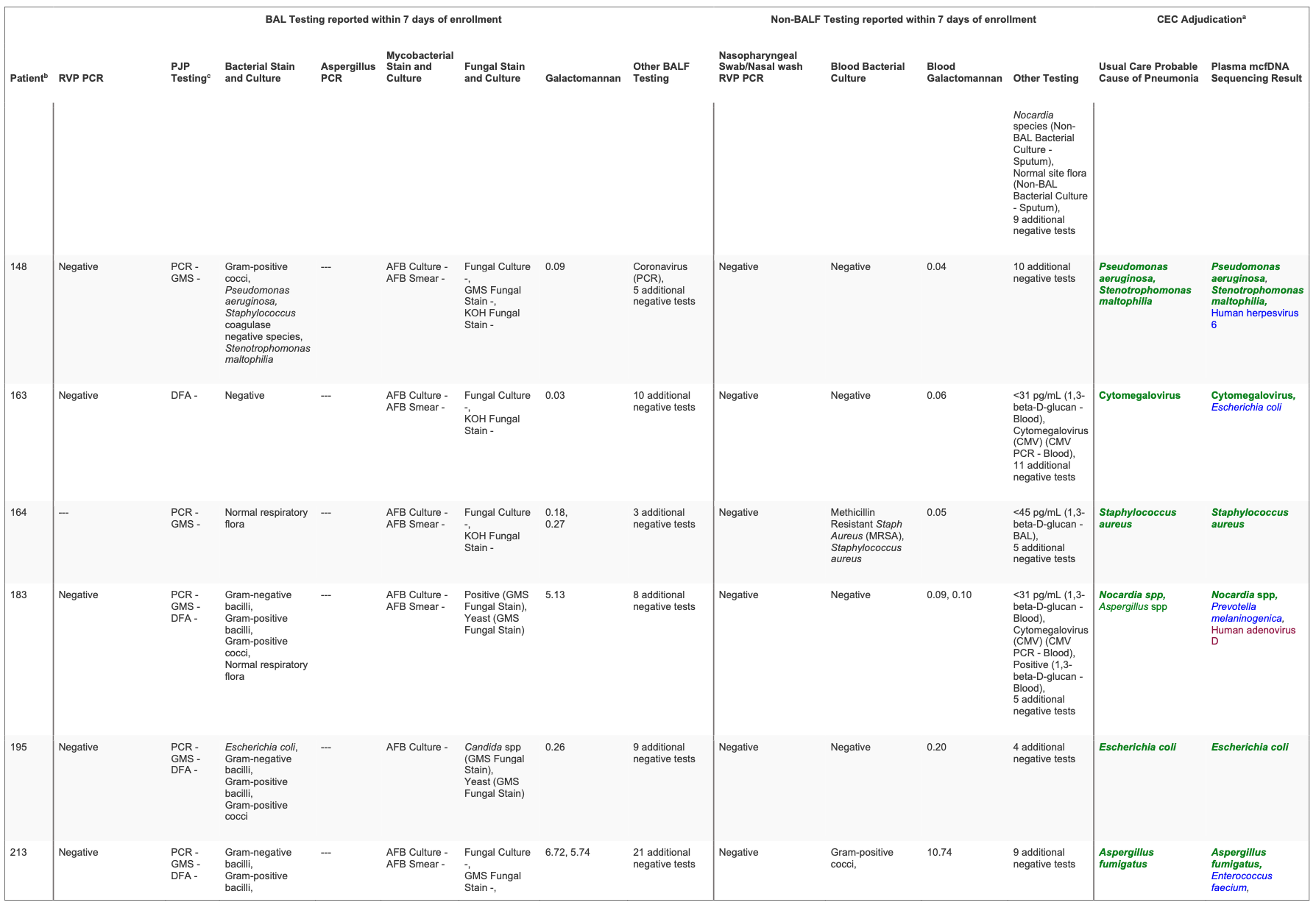


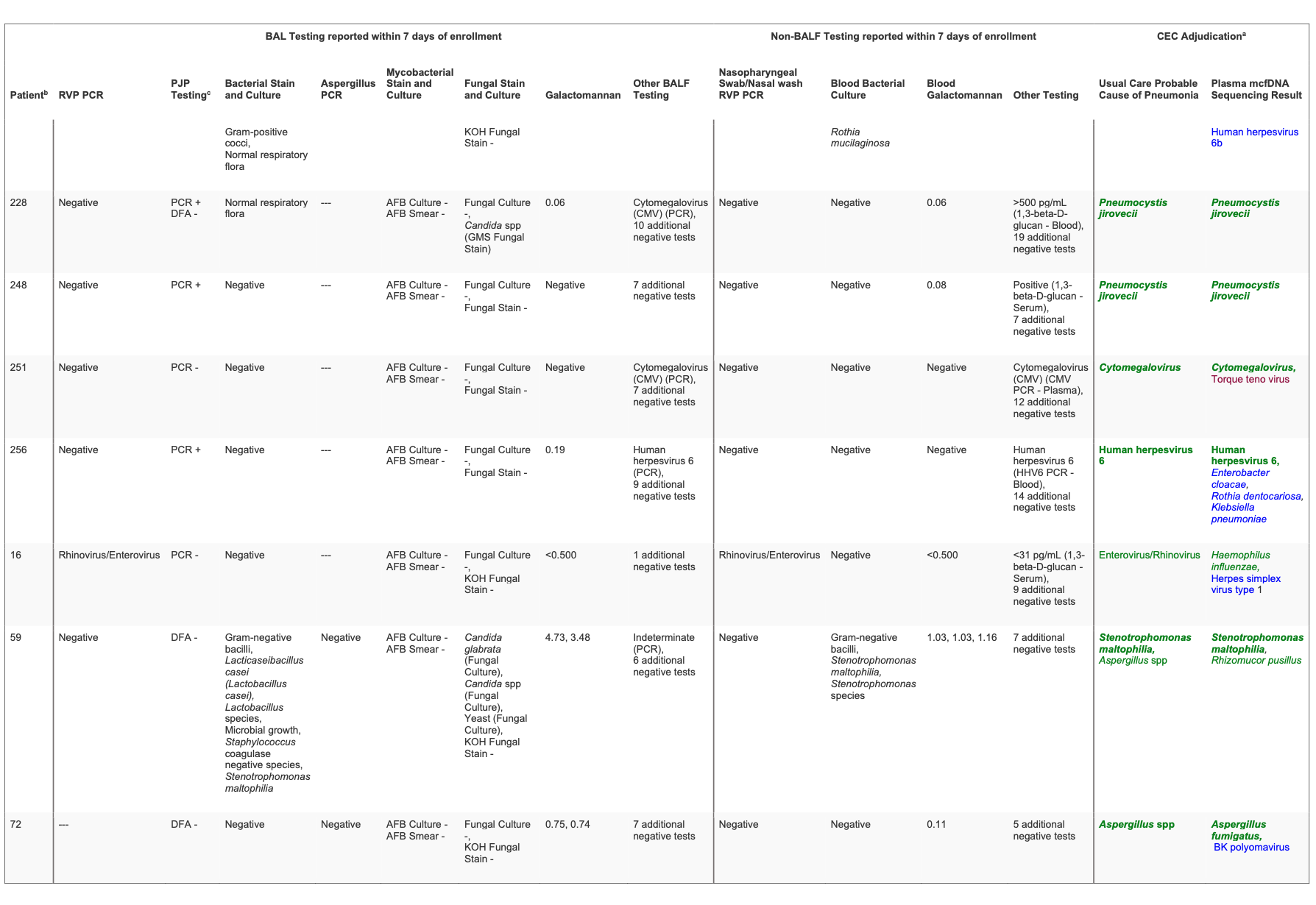


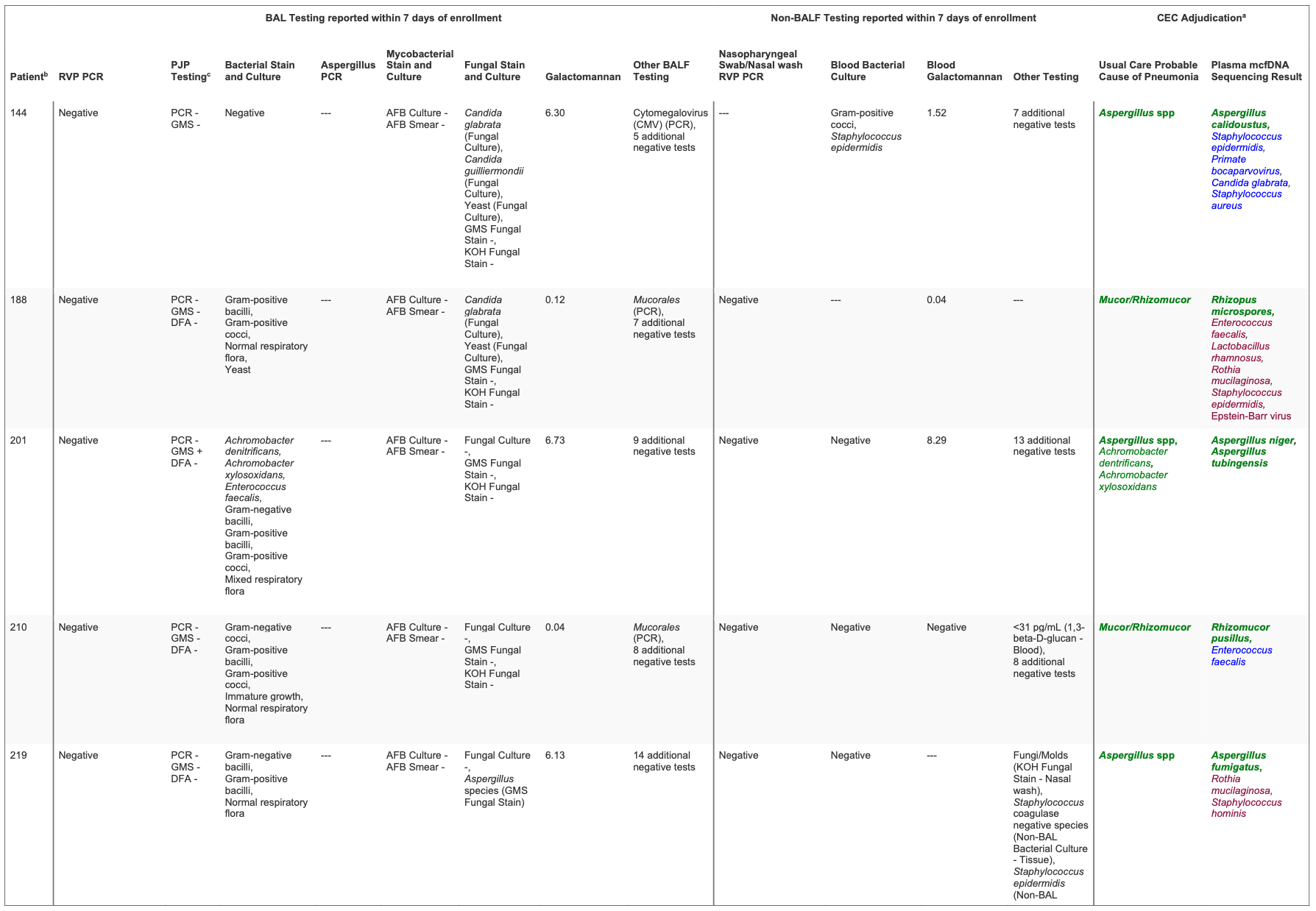


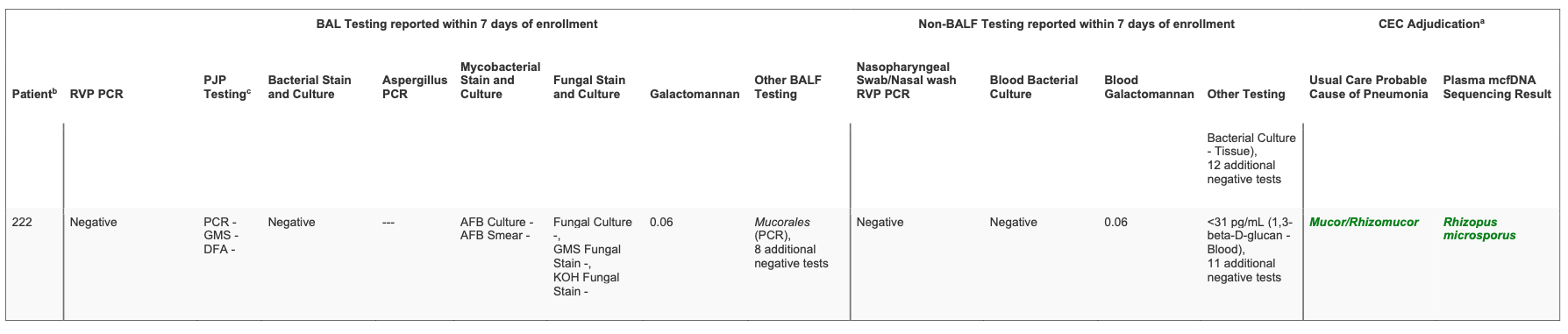


An interactive online dashboard that includes all raw diagnostic testing data for the entire Per Protocol population is available. Instructions for accessing the interactive dashboard are available in the Online Supplement.

^a^ Adjudication classifications are presented by color. Microbe names in green text were adjudicated as a probable cause of pneumonia; blue, a clinically relevant infection other than pneumonia; red, not causing an active infection (commensal organism or contaminant). Bold text represents a match between Usual Care and plasma mcfDNA sequencing test results.

^b^ Patients are presented in the following order: 1) Probable cause of pneumonia exclusively identified by plasma microbial cell-free DNA sequencing 2) Probable cause of pneumonia exclusively identified by usual care testing 3) Same probable cause of pneumonia identified by both usual care and plasma microbial cell-free DNA sequencing. Patients with no cause of pneumonia identified are not presented.

^c^ Includes only staining results for Pneumocystis jirovecii identification. Other fungal organisms identified by Grocott methenamine silver staining are included in the column entitled Fungal Stain and Culture.

^d^ Serratia marcescens was isolated from respiratory cultures 10 days before study enrollment. The patient was enrolled upon undergoing bronchoscopy, as the clinical team was not certain this organism was the cause of persistent pneumonia. Clinical Events Committee determined persistent infection with previously isolated Serratia marcescens was the probable cause of persistent pneumonia identified by usual care before plasma microbial cell-free DNA sequencing results were unblinded. This organism is not presented in usual care testing results as the positive culture resulted 10 days before enrollment.

Abbreviations: ---, test not performed; AFB, acid-fast bacillus; BALF, bronchoalveolar lavage fluid; CEC, Clinical Events Committee; DFA, direct fluorescent antibody; GM, galactomannan; GMS, Grocott methenamine silver; KOH, potassium hydroxide; NP, nasopharyngeal; PCR, polymerase chain reaction; RVP, respiratory viral panel.

eTable 2. Diagnostic Testing Results for Patients with Discordant Fungal Pneumonia Diagnoses in the Per Protocol Population.

|  | **Usual Care Testing Reported within 7 Days of Enrollment** | | | | **Post-Hoc BALF PCR Testing**^b^ | | | **CEC Adjudication** | |
| --- | --- | --- | --- | --- | --- | --- | --- | --- | --- |
| **Patient**^a^ | **BALF**  **Fungal PCR** | **BALF Fungal Stain and Culture** | **BALF GM** | **Blood GM** | **Pan *Aspergillus*** | ***Aspergillus Fumigatus*** | ***Aspergillus Terreus*** | **Usual Care Probable Cause of Pneumonia** | **Plasma mcfDNA Sequencing Result** |
| 27 | --- | Fungal Culture - KOH Fungal Stain - | 0.20 | 0.20, 0.15 | Positive | Negative | Negative |  | *Aspergillus fumigatus* |
| 52 | *Aspergillus*  Negative | Fungal Culture - KOH Fungal Stain - | 0.25 | 0.10 | Positive | Negative | Negative |  | *Cunningham-ella* |
| 179^c^ | *Mucorales* negative | *Candida* spp (GMS Fungal Stain) Positive (GMS Fungal Stain) Yeast (GMS Fungal Stain) | 0.08 | 0.05 | Negative | Negative | Negative |  | *Rhizomucor pusillus* |
| 154^d^ | --- | KOH Fungal Stain - | 0.49 | 0.1, 0.12 | N/A | N/A | N/A |  | *Aspergillus fumigatus* |
| 48 | *Aspergillus*  Negative | Candida albicans (Fungal Culture) Yeast (Fungal Culture) KOH Fungal Stain - | 3.91, 3.54 | 0.04 | Negative | Negative | Negative | *Aspergillus* spp |  |
| 49 | *Aspergillus*  Negative | Fungal Culture - KOH Fungal Stain - | 2,  1.55 | 0.03 | Negative | Negative | Negative | *Aspergillus* spp |  |
| 55 | *Aspergillus nidulans* | Fungal Culture - KOH Fungal Stain - | 4.13, 3.26 | 0.04 | Positive | Negative | Negative | *Aspergillus* spp |  |
| 65 | *Aspergillus*  Negative | Fungal Culture - KOH Fungal Stain - | 2.42, 2.36 | 0.05, 0.06, 0.10, 0.12, 0.04 | Negative | Negative | Negative | *Aspergillus* spp |  |
| 67 | *Aspergillus fumigatus* | Fungal Culture - KOH Fungal Stain - | 0.05, 0.03 | 4.13, 4.84 | Positive | Positive | Negative | *Aspergillus fumigatus* |  |
| 70 | *Aspergillus fumigatus* | Few fungal elements (KOH Fungal Stain) | 0.70 | 0.50, 0.56 | Negative | Negative | Negative | *Aspergillus fumigatus* |  |
| 83 | --- | Fungal Culture - GMS Fungal Stain - | 8.94 | 0.25 | Positive | Positive | Negative | *Aspergillus* spp |  |
| 120 | *Aspergillus*  Negative | GMS Fungal Stain - KOH Fungal Stain - | 8.13 | 1.05, 1.33 | Inconclusive | Positive | Negative | *Aspergillus* spp |  |
| 141 | *Aspergillus*  Negative | Fungal Culture - GMS Fungal Stain - KOH Fungal Stain - | 0.65 | 0.05 | Negative | Negative | Negative | *Aspergillus* spp |  |
| 196 | *Mucorales* negative | *Aspergillus* spp (GMS Fungal Stain) Yeast (KOH Fungal Stain) | 0.69 | 0.01 | Positive | Positive | Negative | *Aspergillus* spp |  |
| 202 | *Mucorales* negative | Fungal Culture - GMS Fungal Stain - KOH Fungal Stain - | 0.33 | 0.14 | Negative | Negative | Negative | *Aspergillus* Spp |  |
| 215 | *Mucorales* negative | Fungal Culture - GMS Fungal Stain - KOH Fungal Stain - | 3.97 | 0.05, 0.03 | Negative | Negative | Negative | *Aspergillus* spp |  |
| 169^d^ | *Aspergillus*  Negative | KOH Fungal Stain - | 0.61, 0.18 | 0.05 | N/A | N/A | N/A | *Aspergillus* spp |  |
| 180^d^ | *Mucorales* negative | GMS Fungal Stain - KOH Fungal Stain - | 0.94 | 0.04, 0.05 | N/A | N/A | N/A | *Aspergillus* spp |  |

^a^ Patients are presented in the following order: 1) Fungal cause of pneumonia exclusively identified by plasma microbial cell-free DNA sequencing 2) Fungal cause of pneumonia exclusively identified by usual care testing. Post-hoc PCR testing was not performed in cases where usual care and plasma microbial cell-free DNA sequencing identified the same cause of fungal pneumonia.

^b^ Post-hoc polymerase chain reaction testing with the Eurofins Viracor (Lenexa, Kansas) Aspergillus PCR Panel was performed in all patients with a mold identified as a probable cause of pneumonia by either usual care testing or microbial cell-free DNA sequencing if the patient consented to future research testing of study samples and remnant bronchoalveolar lavage fluid was available. This PCR panel includes three real-time PCR assays (Pan-*Aspergillus, Aspergillus fumigatus,* and *Aspergillus terreus*).

^c^ Post-hoc PCR testing for Mucorales was performed as this organism was exclusively identified by plasma microbial cell-free DNA sequencing in this patient. PCR testing was positive for Mucorales.

^d^ Patient did not consent to future research testing of study samples.

Abbreviations: ---, test not performed; BALF, bronchoalveolar lavage fluid; CEC, Clinical Events Committee; GM, galactomannan; GMS, Grocott methenamine silver; KOH, potassium hydroxide; PCR, polymerase chain reaction.

eTable 3. Adjudicated Possible Changes in Antimicrobial Therapy Among Patients with a Pneumonia Etiology Exclusively Identified by Plasma mcfDNA Sequencing.

| **≥ Adjudicated potential pneumonia antimicrobial changes**, No. (%)^a,b^ | 17/21 (81) |
| --- | --- |
|  | |
| **Potential antibacterial therapy changes,** No. (%) | 12/17 (71) |
| Broaden antibacterial coverage | 7/12 (58) |
| Additional aerobic Gram-negative coverage | 1 |
| Additional anaerobic coverage | 3 |
| Other | 3 |
|  | |
| Narrow antibacterial coverage | 5/12 (42) |
| Stopped ≥1 Gram-negative agent | 1 |
| Stopped MRSA coverage | 2 |
| Stopped MRSA coverage and ≥1 Gram-negative agent | 1 |
| Stopped MRSA coverage and ≥1 anaerobic agent | 1 |
|  | |
| Earlier antibacterial coverage | 1/12 (8) |
|  | |
| **Potential antiviral therapy changes,** No. (%) | 1/17 (6) |
| Added ≥1 agent | 1 |
|  | |
| **Potential antifungal coverage,** No. (%) | 5/17 (29) |
| Added ≥1 agent | 2 |
| Added ≥1 agent and stopped ≥1 agent | 1 |
| Stopped ≥1 agent | 2 |

^a^ Clinical Events Committee adjudicated potential antimicrobial changes had plasma microbial cell-free DNA sequencing test results been available in real time. Potential antimicrobial changes were only adjudicated in cases where plasma microbial cell-free DNA sequencing identified a probable cause of pneumonia. Potential antimicrobial changes were not adjudicated when plasma microbial cell-free DNA sequencing was negative.

^b^ The Clinical Events Committee adjudicated more than 1 antimicrobial change was possible for some patients.

Abbreviation: MRSA, methicillin-resistant *Staphylococcus aureus.*

eTable 4. Usual Care Testing, Potential Antimicrobial Changes, and Mortality in Patients with a Pneumonia Etiology Exclusively Identified by Plasma Microbial Cell-Free DNA Sequencing.

| **Patient Identifier** | **Probable Cause(s) of Pneumonia Identified by Plasma mcfDNA Sequencing** | **Usual Care Tests for Identified Pathogen** | **Adjudicated Potential Antimicrobial Changes for Pneumonia** | **Vital Status at 30 days** |
| --- | --- | --- | --- | --- |
| 27 | *Aspergillus fumigatus* | Fungal Culture – BALF Galactomannan – Serum (0.2)  Galactomannan – BALF (0.2) | None | Alive |
| 32 | *Nocardia cyriacigeorgica* | AFB Culture – BALF  AFB Stain – BALF  Bacterial Culture – Blood  Bacterial Culture – BALF  Bacterial Stain – BALF | Broadened antimicrobial coverage: Addition of 2 agents with activity against *Nocardia* spp | Alive |
| 52 | *Cunninghamella* | Fungal Culture – BALF  KOH Fungal Stain – BALF  Fungal PCR – BALF  Fungal PCR – Tissue  Zygomycete PCR – Tissue  Zygomycete PCR – BALF | Antifungal coverage change: Added >= 1 agent;  Stopped >= 1 agent | Deceased |
| 56 | *Rothia* species | Bacterial Culture – Blood  Bacterial Culture – BALF  Bacterial Stain – BALF | Antifungal coverage change: Stopped >= 1 agent | Alive |
| 74 | *Prevotella melaninogenica;*  *Rothia mucilaginosa* | Bacterial Culture – Blood  Bacterial Culture – BALF  Bacterial Stain – BALF | Broadened antimicrobial coverage: Additional anaerobic coverage; Earlier coverage | Alive |
| 76 | *Rothia mucilaginosa; Streptococcus oralis* | Bacterial Culture – Blood  Bacterial Culture – BALF  Bacterial Stain – BALF | None | Alive |
| 105 | *Acinetobacter* species | Bacterial Culture – Blood  Bacterial Culture – BALF  Bacterial Stain – BALF | Broadened antimicrobial coverage: Additional aerobic Gram-negative coverage | Alive |
| 108 | *Legionella hackeliae* | Bacterial Culture – Blood  Bacterial Culture – BALF  Bacterial Stain – BALF  Legionella Antigen – Urine | None | Alive |
| 114 | *Prevotella melaninogenica* | Bacterial Culture – Blood  Bacterial Culture – BALF  Bacterial Stain – BALF | Broadened antimicrobial coverage: Additional anaerobic coverage | Alive |
| 124 | *Pseudomonas aeruginosa* | Bacterial Culture – Blood  Bacterial Culture – BALF  Bacterial Stain – BALF | Narrowed antimicrobial coverage: Stopped MRSA coverage,  Stopped >= 1 anaerobic agent | Alive |
| 138 | *Klebsiella pneumoniae* | Bacterial Culture – Blood  Bacterial Culture – BALF  Bacterial Stain – BALF | Narrowed antimicrobial coverage: Stopped MRSA coverage | Alive |
| 140 | *Pseudomonas aeruginosa* | Bacterial Culture – Blood  Bacterial Culture – BALF  Bacterial Stain – BALF | Narrowed antimicrobial coverage: Stopped MRSA coverage | Alive |
| 154 | *Aspergillus fumigatus* | Fungal Culture – BALF  KOH Fungal Stain – BALF,  Galactomannan – Serum (0.1, 0.12, 0.2, 0.61)  Galactomannan – BALF (0.49) | Antifungal coverage change: Added >= 1 agent | Deceased |
| 161 | *Legionella micdadei* | Bacterial Culture – Blood  Bacterial Culture – BALF  Bacterial Stain – BALF | Broadened antimicrobial coverage: Add atypical coverage with either fluoroquinolone or macrolide for *Legionella* | Alive |
| 170 | *Pneumocystis jirovecii* | Fungal Culture – BALF  Beta-D-glucan – Serum  GMS PJP Stain – BALF | Broadened antimicrobial coverage: Add *Pneumocystis* treatment | Alive |
| 175 | *Pneumocystis jirovecii* | Fungal Culture – BALF  Beta-D-glucan – Serum (198 pg/mL)  GMS PJP Stain – BALF | Antifungal coverage change: Added >= 1 agent | Alive |
| 179 | *Mucor/Rhizomucor; Rhizomucor pusillus* | Fungal Culture – Pleural Fluid  Fungal Culture – BALF  Fungal Stain – Pleural Fluid  GMS Fungal Stain – BALF  KOH Fungal Stain – BALF  Mucorales PCR – BALF  Universal PCR – Pleural Fluid | Narrowed antimicrobial coverage: Stopped MRSA coverage,  Stopped >= 1 Gram negative agent | Alive |
| 182 | Human Herpesvirus 6 | Viral Culture – BALF | Antiviral coverage change:  Added >= 1 agent | Alive |
| 197 | *Pneumocystis jirovecii* | Fungal Culture – BALF  GMS Fungal Stain – BALF  Fungitell Assay – Blood  DFA PJP Stain – BALF  GMS PJP Stain – BALF  PJP PCR – BALF | None | Deceased |
| 217 | *Prevotella melaninogenica; Streptococcus infantus* | Bacterial Culture – Blood  Bacterial Culture – BALF  Bacterial Stain – BALF | Broadened antimicrobial coverage: Additional anaerobic coverage | Alive |
| 252 | *Legionella anisa* | Bacterial Culture – Blood  Bacterial Culture – BALF  Bacterial Stain – BALF  Legionella Antigen – Urine | Narrowed antimicrobial coverage: Stopped >= 1 Gram negative agent | Alive |

^a^ Completed usual care tests capable of identifying the adjudicated probable cause of pneumonia are included.

Abbreviation: AFB, acid-fast bacillus; BALF, bronchoalveolar lavage fluid; DFA, direct fluorescent antibody; GMS, Grocott methenamine silver; KOH, potassium hydroxide; mcfDNA, microbial cell-free DNA, PCR, polymerase chain reaction; PJP, *Pneumocystis jirovecii.*

eTable 5. Clinically Relevant Non-Pneumonia Infections Identified by Plasma Microbial Cell-Free DNA Sequencing (Per Protocol Population ^a^).

| **≥1 non-pneumonia infection identified by plasma mcfDNA sequencing,** No. (%) | 67/173 (39) |
| --- | --- |
|  | |
| **Sites of infection,** No.^b^ |  |
| Bloodstream | 21 |
| Latent Viral Reactivation | 20 |
| Gastrointestinal Translocation | 14 |
| Unknown^c^ | 8 |
| Genitourinary tract | 4 |
| Skin or skin structures | 4 |
| Intra-abdominal/gastrointestinal | 3 |
| Bone and joint | 1 |
| Endovascular | 1 |
| Perirectal abscess | 1 |

^a^ Clinical Events Committee adjudicated the clinical significance of all microbes identified on the plasma microbial cell-free DNA sequencing test report. If a microbe was not adjudicated as a probable cause of the patient’s index pneumonia, adjudicators determined if the microbe was causing another type of active infection. This table summarizes the primary sites of active non-pneumonia infections identified by plasma microbial cell-free DNA sequencing tests collected within 1 day of study enrollment.

^b^ More than one adjudicated non-pneumonia infection was identified in some patients.

^c^ Pathogens adjudicated by the Clinical Events Committee as a likely cause of clinically significant infection likely requiring antimicrobial therapy, but the primary site of infection could not be determined.

Abbreviation: mcfDNA, microbial cell-free DNA.

eTable 6. Measures of Agreement Between Usual Care Testing and Plasma Microbial Cell-Free DNA Sequencing – Fungal Etiologies Only (Per Protocol Population^a^).

|  | | **All Usual Care Testing** | | |
| --- | --- | --- | --- | --- |
|  |  | **Positive** | **Negative** | **Total** |
| **Plasma mcfDNA sequencing** | **Positive** | 13 | 7 | 20 |
|  | **Negative** | 17 | 136 | 153 |
|  | **Total** | 30 | 143 | 173 |
|  | | | | |
| **Measure** | **Percent (95% confidence limits**^b^**)** | | | |
| Positive percent agreement | 43.3 (25.5, 62.6) | | | |
| Negative percent agreement | 95.1 (90.2, 98.0) | | | |

^a^ The Per Protocol Population included patients with complete protocol-required testing, a valid plasma mcfDNA sequencing test collected within 24 hours of enrollment, and no protocol deviations.

^b^ Clopper-Pearson confidence intervals

Abbreviation: mcfDNA, microbial cell-free DNA.

eTable 7. Measures of Agreement Between Usual Care Testing and Plasma Microbial Cell-Free DNA Sequencing – Bacterial Etiologies Only (Per Protocol Population^a^).

|  | | **All Usual Care Testing** | | |
| --- | --- | --- | --- | --- |
|  |  | **Positive** | **Negative** | **Total** |
| **Plasma mcfDNA sequencing** | **Positive** | 11 | 14 | 25 |
|  | **Negative** | 7 | 141 | 148 |
|  | **Total** | 18 | 155 | 173 |
|  | | | | |
| **Measure** | **Percent (95% confidence limits**^b^**)** | | | |
| Positive percent agreement | 61.1 (35.7, 82.7) | | | |
| Negative percent agreement | 91.0 (85.3, 95.0) | | | |

^a^ The Per Protocol Population included patients with complete protocol-required testing, a valid plasma mcfDNA sequencing test collected within 24 hours of enrollment, and no protocol deviations.

^b^ Clopper-Pearson confidence intervals

Abbreviation: mcfDNA, microbial cell-free DNA.

eTable 8. Measures of Agreement Between Usual Care Testing and Plasma Microbial Cell-Free DNA Sequencing – Viral Etiologies Only (Per Protocol Population^a^).

|  | | **All Usual Care Testing** | | |
| --- | --- | --- | --- | --- |
|  |  | **Positive** | **Negative** | **Total** |
| **Plasma mcfDNA sequencing**^b^ | **Positive** | 3 | 1 | 4 |
|  | **Negative** | 4^c^ | 165 | 169 |
|  | **Total** | 7 | 166 | 173 |
|  | | | | |
| **Measure** | **Percent (95% confidence limits**^d^**)** | | | |
| Positive percent agreement | 42.9 (9.9, 81.6) | | | |
| Negative percent agreement | 99.4 (96.7, 100.0) | | | |

^a^ The Per Protocol Population included patients with complete protocol-required testing, a valid plasma mcfDNA sequencing test collected within 24 hours of enrollment, and no protocol deviations.

^b^ Plasma mcfDNA sequencing does not detect RNA viral pathogens.

^c^ Includes 4 RNA viruses identified by usual care testing and adjudicated as probable cause of pneumonia.

^d^ Clopper-Pearson confidence intervals

Abbreviation: mcfDNA, microbial cell-free DNA.
